# A Review of Western Australian Researchers’ Contributions to Understanding Cancer Prevention and Outcomes in Aboriginal People

**DOI:** 10.3390/ijerph23060777

**Published:** 2026-06-10

**Authors:** Veisinia Pulu, Emma V. Taylor, Phuntsho Om, Sandra C. Thompson

**Affiliations:** Western Australian Centre for Rural Health (WACRH), University of Western Australia, P.O. Box 109, Geraldton, WA 6531, Australia; emma.taylor@uwa.edu.au (E.V.T.); phuntsho.om@uwa.edu.au (P.O.); sandra.thompson@uwa.edu.au (S.C.T.)

**Keywords:** Indigenous, cancer disparities, cancer support, cancer screening, cultural safety, culturally sensitive care, Aboriginal workforce, community engagement, Western Australia, patient navigators, co-design, telehealth, equity

## Abstract

**Highlights:**

**Public health relevance—How does this work relate to a public health issue?**

- Cancer is a leading cause of death among Aboriginal people in Australia, with ongoing disparities in incidence, mortality, and survival outcomes compared to non-Aboriginal populations.
- This review addresses core public health concerns by examining prevention, screening participation, access to care, and survival outcomes in Western Australia, directly addressing health inequities and social determinants of health.

**Public health significance—Why is this work of significance to public health?**

- This review synthesizes decades of regional research to identify systemic, cultural, geographic, and socioeconomic barriers contributing to cancer disparities, strengthening the evidence base for reducing inequities.
- By highlighting gaps in prevention, early detection, treatment access, and survivorship, this work supports more equitable health service planning and resource allocation.

**Public health implications—What are the key implications or messages for practitioners, policy makers and/or researchers in public health?**

- Practitioners and policymakers must prioritize culturally secure, community-led cancer prevention and screening initiatives, and improve access to timely, appropriate treatment for Aboriginal people, particularly in rural and remote areas.
- Researchers should continue strengthening Indigenous data quality, embed Indigenous governance in research, and focus on implementation studies that translate evidence into measurable improvements in cancer outcomes.

**Abstract:**

Aboriginal people in Western Australia (WA) experience poorer cancer outcomes compared to non-Aboriginal Australians, with significant disparities in cancer screening participation, later-stage diagnosis, and lower survival rates. This narrative review, informed by selected scoping methods, examined 69 peer-reviewed studies contributed by WA researchers from 2000 to 2024 to inform understanding of and address these inequities. Recurring issues requiring attention included promoting cultural safety in healthcare, addressing barriers to and disparities in cancer care, boosting cancer screening and awareness, enhancing education and communication, strengthening support systems and care navigation, improving treatment access and outcomes, and building workforce capacity. Recommendations to address the above challenges and improve cancer care and outcomes for Aboriginal people in WA included addressing barriers and disparities in cancer care; promoting effective education, communication, and culturally appropriate support; enhancing cancer screening participation and awareness initiatives; improving access to cancer treatment and outcomes; strengthening policy and system-level interventions; supporting families and communities throughout their cancer journey; building research capacity and data collection to guide Aboriginal and community-led initiatives. These recommendations highlighted that multi-level interventions are needed, from empowering Aboriginal people and strengthening communities to improving service delivery and driving systematic reforms. Overall, this narrative review informs future research, policy, and practice focused on equity to improve cancer outcomes for Aboriginal people in WA and beyond.

## 1. Introduction

Cancer remains a leading cause of death among Aboriginal and Torres Strait Islander (hereafter we respectfully use the term Aboriginal) people in Australia, with ongoing disparities in incidence, mortality, and survival outcomes compared to non-Aboriginal populations. Research shows that Aboriginal people are more likely to be diagnosed with cancer at later stages and experience lower survival rates compared to non-Aboriginal populations [1,2,3]. For example, in Western Australia (WA), Aboriginal people are disproportionately diagnosed with cancer at more advanced stages and often experience a 20–30% higher five-year mortality rate following diagnosis compared to their non-Aboriginal counterparts. These outcomes reflect both intrinsic (biomedical) and extrinsic (social, cultural, and systemic inequities) factors that influence access to screening, early detection, treatment, and supportive cancer care [4].

Various interconnected barriers contribute to this disparity, including geographical isolation, financial hardship, cultural gaps between service delivery and community expectations, and limited representation of Aboriginal people in the health workforce and decision-making processes. WA is geographically vast, with much of the population living outside metropolitan areas. Aboriginal people are more likely to live in rural and remote areas and tend to experience social and economic challenges and limited access to health services, compared to non-Indigenous Australians [5]. Moreover, Aboriginal people living in rural areas often need to travel vast distances to access cancer treatment services; this imposes additional emotional and financial strain on individuals and families [6]. Furthermore, mainstream services often lack the cultural safety and responsiveness essential for effectively engaging Aboriginal people and communities, resulting in mistrust and diminished participation in screening and treatment processes [7,8,9,10].

In 2025, Cancer Council Western Australia commissioned a narrative review to assess research published between 2000 and 2024 which centred around cancer experiences for Aboriginal people in a WA context, and the content of this review is reported here. The review aimed to: (1) examine existing knowledge and past recommendations aimed at improving cancer outcomes for Aboriginal communities in WA; and (2) inform future research, policy, and investment priorities that centre around Aboriginal voices and knowledge systems. This study reports on and critically examines Aboriginal cancer research from WA-based researchers. This approach recognizes that some of this research was led outside of WA, focused on health services research or activities which were undertaken elsewhere, had received some funding support from Cancer Council WA, and can provide valuable learnings to inform future research efforts and funding approaches within WA. The review examined cancer across the spectrum, from prevention to palliative care, and includes experiences and outcomes among Aboriginal people in WA, support for Aboriginal people with cancer, disparities in cancer outcomes, and recommendations from these studies. The information gathered can help identify what is needed to reduce inequities in cancer care and guide future priorities in treatment, policy development, and research funding.

## 2. Materials and Methods

This narrative review utilized selected scoping review methods to examine contributions of WA-based researchers from 2000 to August 2024. To identify relevant literature, searches were undertaken in PubMed, CINAHL, the Cochrane Library, Australian Indigenous HealthInfoNet, and Google Scholar. The search approach combined MeSH headings with key search terms such as “Aboriginal,” “cancer,” “Western Australia,” “screening,” “treatment,” and “support.” (see Supplementary File S1) The inclusion criteria included: (1) studies published in English between 2000 and 2024; (2) a focus on Aboriginal and Torres Strait Islander peoples in WA or inclusion of WA-based researchers; and (3) relevance to any part of the cancer care continuum (Table 1). All studies identified were checked to ensure that the research was undertaken in WA or had an author affiliated with a WA institution.

A defining criterion for this review was the inclusion of research led by, or involving, Western Australian-affiliated researchers. We acknowledge that researcher affiliation does not always strictly correlate with a study’s geographic focus; however, this parameter was selected to highlight the specific contributions and influence of the WA research community on the national discourse regarding Aboriginal cancer outcomes. This approach provides unique insight into how local research expertise is being leveraged to address health disparities, regardless of whether the primary data collection occurred solely within state borders.

### 2.1. Data Synthesis and Analysis

The analysis utilized an inductive thematic synthesis to identify recurring priorities within the 69 included studies. To ensure methodological rigour, the review was managed via Covidence, utilizing a two-stage independent screening protocol. Titles and abstracts were reviewed independently by three researchers (NP, PO and ST). Subsequently, two researchers (NP, PO) independently assessed all full-text studies before their results were shared with the rest of the team. As shown in Table 1, studies were excluded following full-text review if they did not focus on Aboriginal populations, were unrelated to cancer, or were not published in peer-reviewed journals. Any discrepancies were resolved through team discussion to reach 100% consensus before extraction. A structured extraction template captured study characteristics, populations, cancer focus, methodology, main findings and recommendations.

### 2.2. Coding Procedure

Two authors (NP and PO) reviewed the recommendations from each study alongside their main findings and grouped them into key themes. The broader research team then discussed, refined, and finalized these themes to ensure they were coherent and reflected the evidence. Recommendations were extracted using an open coding process, where action-oriented statements from each paper were documented in a standardized matrix. These codes were then iteratively grouped into broader descriptive themes. This process was not restricted by a pre-defined theoretical framework, allowing the seven key areas to emerge organically from the data as a grounded reflection of the WA research landscape. As the study relied on published literature already in the public domain, ethics approval was not required.

## 3. Results

**Figure 1:**
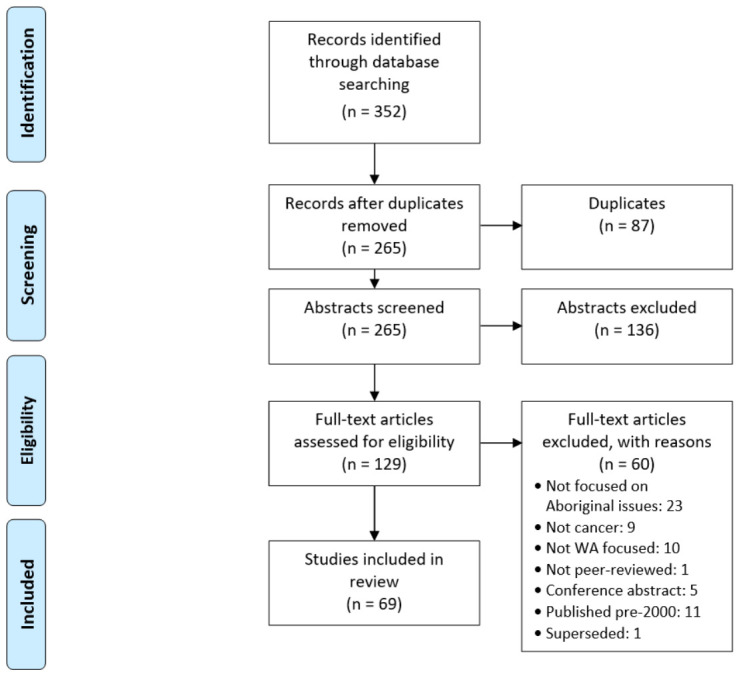
Summary of the process of identification, screening, and inclusion of studies.

We identified 69 studies that met our inclusion criteria. The results for each stage of our search and screening processes are shown in the flow diagram (Figure 1).

### 3.1. Study Characteristics

Of the 69 publications, 28 (40%) used qualitative methods (summary available in Supplementary File S2); 28 (40%) used quantitative methods (epidemiological and survey type research) (Supplementary File S3); 10 were reviews (17%) (Supplementary File S4); and 3 studies used mixed methods (Supplementary File S5).

The majority (39; 57%) focused specifically on WA, 5 (7%) studies included WA and one or more other states, and 25 (36%) studies reported research from across Australia. The most studied populations were Aboriginal people with cancer 49 (71%), with additional studies including caregivers, health professionals, and broader population groups. Most of the included studies were conducted in WA.

### 3.2. Focus Areas of Research

Key research focus areas were barriers and disparities in cancer care (*n* = 18), education and communication (*n* = 18), cancer screening and prevention (*n* = 13), incidence and mortality (*n* = 11), and cancer treatment and outcomes (*n* = 9). Only 13 studies described or evaluated an intervention; the majority were observational. Most interventions were educational (*n* = 6) or medical (*n* = 4). Additional types included supportive care (*n* = 2), preventive measures (*n* = 1), lifestyle-focused interventions (*n* = 1), community and social approaches (*n* = 1), and two classified as “other” (see Table 2).

Across the reviewed studies, most publications did not specify an age group (37), followed by studies focusing on adults aged 24–64 (27) and the elderly aged 65 and over (20). Research involving young adults aged 18–24 accounted for 13 publications, while children aged 0–18 were the least represented group with only 8 publications, as shown in Table 3.

### 3.3. Key Areas Identified

Analysis of the 69 studies identified seven key areas that were reported on with respect to the challenges and opportunities for improving Aboriginal cancer care outcomes. A summary of key recommendations derived from the included studies for each of these areas is reported in Table 4.

### 3.4. Summary of Recommendations

Analysis of the studies revealed that majority of the recommendations centred on the following synthesized key areas: addressing barriers and disparities in cancer care; promoting effective education, communication, and culturally appropriate support; enhancing cancer screening participation and awareness initiatives; improving access to cancer treatment and outcomes; strengthening policy and system-level intervention; supporting families and communities throughout their cancer journey; building research capacity and data collection to guide Aboriginal and community-led initiatives. These recommendations were further distilled into core priorities: embedding Aboriginal health priorities in policy, expanding Aboriginal-led support networks, tailoring screening initiatives, integrating Aboriginal Patient Navigators (APNs) and Aboriginal Liaison Officers (ALOs) into care teams, and strengthening Aboriginal participation in governance and research. Telehealth and home-based care were also identified as effective strategies to improve access in remote areas. Together, these recommendations emphasized a holistic, community-led approach that integrates policy, service delivery, and culturally grounded practices, promoting culturally safe, accessible, and responsive cancer care and ultimately contributing to improved outcomes and reduced inequities for Aboriginal populations. The discussion section further elaborates on and explores these core priorities to guide future research, policy, and investment strategies that centre Aboriginal voices and knowledge systems.

## 4. Discussion

This narrative review highlights the substantial and ongoing contributions of Western Australian researchers towards understanding the cancer care experiences and outcomes of Aboriginal people. Despite this substantial research, growing evidence and numerous recommendations over time, a critical gap remains in translating this knowledge into sustained systemic change. While there has been progress in cancer service delivery, Aboriginal people in WA continue to experience poorer outcomes along the cancer care continuum, with late-stage diagnoses, lower participation in screening, and reduced access to culturally safe and timely treatment options. These outcomes reflect persistent concerns raised by Aboriginal communities, healthcare providers, and researchers, which reinforce the need for systematic change and culturally responsive care models.

The seven key areas identified in this review were summarized into core priorities and included improving Aboriginal participation in governance and research. Collectively, the core priorities underscore the importance of cultural safety in cancer care for Aboriginal communities. However, essential cultural safety remains inconsistently applied in clinical settings [17,23]. The integration of Aboriginal Liaison Officers (ALOs) and Aboriginal Patient Navigators (APNs) into care teams was a tangible, effective strategy to build trust, improve communication, and enhance continuity of care [12,13].

Structural and systemic barriers such as long travel distances, financial hardship, poor health literacy, and fragmented service systems continue to impede Aboriginal people’s access to timely cancer care and are particularly difficult to address given the geographic spread of WA and the higher proportion of the Aboriginal population living in more remote areas. Studies noted that many Aboriginal patients are required to relocate for treatment, often leaving behind family and community supports [14,16]. These disruptions contribute to emotional distress and loss of connection to Country. Telehealth emerged as a valuable strategy to reduce logistical burdens, although it must be implemented in culturally appropriate ways with local community involvement [13,14].

Participation in cancer screening programs remains significantly lower for Aboriginal people compared to the non-Aboriginal population. This review found that culturally adapted education materials, Aboriginal-led health promotion campaigns, and integration of screening within primary care settings all contribute to improved engagement. Community co-design and the inclusion of Aboriginal health workers are essential to tailoring national screening programs to local needs. However, advancements in cancer screening such as risk-stratified techniques and targeted outreach are underutilized in Aboriginal health contexts, which represents a key area for investment [20,21,25,27,40].

This review also emphasized the role of effective communication and education. Mistrust in the healthcare system, compounded by language and cultural differences, continues to impact engagement and understanding. Many studies cited the need for plain language, the use of interpreters, and culturally relevant health education resources that reflect Aboriginal perspectives on health, illness, and healing [13,14]. Moreover, training programs tailored for Aboriginal health professionals were shown to increase both workforce capability and confidence, further highlighting the value of Aboriginal-specific health education in workforce development [22,28]. A more recently published narrative review examined the evidence on how education programs work to improve First Nations Australians’ understanding of cancer, and how this leads to more effective use of prevention, screening and treatment services [41]. It confirmed many of the findings we report here around the need for culturally tailored initiatives, co-design and consultation during development, capacity building to create supportive, trusting environments for accessible cancer education, local ownership and empowerment, integration with existing healthcare systems, flexibility and multi-pronged approaches. The authors also noted that many of the efforts at developing cancer health literacy have not been evaluated.

Support systems, particularly those grounded in Aboriginal community structures, were found to be vital to improved outcomes. Aboriginal-led cancer support networks, such as the ‘Midwest Aboriginal Women’s Cancer Support Group’, demonstrated the importance of peer-led, culturally safe spaces where individuals could share their experiences and access emotional and practical assistance [24]. However, they often face insecure funding, limiting their reach and sustainability [24]. Formalizing these support networks and embedding them into health systems would ensure more consistent access to culturally safe care.

Treatment outcomes were influenced not only by the quality and timing of care, but also by a patient’s ability to navigate culturally unfamiliar systems. Holistic care, defined in the Aboriginal context to encompass emotional, spiritual, and community wellbeing, was often lacking in mainstream models. A small number of studies documented the positive impact of integrating traditional healing practices alongside biomedical treatments, underscoring the importance of respecting Aboriginal perspectives of health and offering choices in care pathways [19,29,30].

The need for workforce development, particularly increasing the representation of Aboriginal people in health governance and service delivery, remains a key area of concern. Aboriginal staff bring cultural expertise, act as advocates, and enhance service accessibility; however, Aboriginal people remain underrepresented in health governance roles, and few services have embedded Aboriginal health priorities into organizational strategy [15,34,35]. Alignment with national frameworks such as the ‘Optimal Care Pathway for Aboriginal and Torres Strait Islander People with Cancer’ has been linked with improved outcomes; nonetheless, uptake remains limited [15].

This review identified that many recommendations made more than a decade previously have continued to re-emerge in recent publications. This highlights the challenges with translating evidence into sustained action. Factors contributing to this implementation gap include limited long-term funding, failure in achieving Aboriginal leadership and participation in decision-making, and inadequate mechanisms and attention given to monitoring progress or evaluating outcomes. Addressing these challenges requires a coordinated, multi-sectoral response that centres Aboriginal voices in research, service design, and policy development [12,13,35].

Furthermore, this review reinforces the importance of integrating upstream, culturally grounded interventions that address broader social determinants, such as housing, income, education, and systemic racism, into cancer prevention and care initiatives. Research and data systems must ensure the identification of Aboriginal status to guarantee that disparities are accurately monitored and addressed. Investing in Aboriginal-led research is also critical to developing initiatives that reflect community values, strengths and priorities [1,15,23,24].

Overall, this review points to promising directions. Co-designed education campaigns, Aboriginal-led support groups, the use of telehealth to support care when appropriate, and evidence-based workforce development initiatives provide a foundation for accessible and effective change. However, to be successful, these approaches must be adequately funded, embedded within policy, and guided by the expertise of Aboriginal communities.

## 5. Conclusions

This narrative review underscores the extensive knowledge base available to guide the efforts of policy makers, service providers and funders in addressing disparities in cancer outcomes for Aboriginal people. The repetition of many findings and recommendations over time highlights the challenges that exist when attention is not centred on sustained, focused actions. There is a significant need for long-term investment in culturally safe, community-led, and system-wide approaches rather than piecemeal approaches that consume resources but fail to achieve sustained change. Key areas for focus include the co-design and delivery of targeted cancer education campaigns, strengthening the Aboriginal health workforce to provide cancer-related services, and supporting service innovations that streamline referral and treatment pathways. Investments in these priorities are essential to improving cancer outcomes and ensuring equitable access to cancer prevention, treatment, and supportive care for Aboriginal communities. We argue for a shift from isolated interventions to sustained, community-led, and system-level responses. Embedding cultural safety in health services delivery, strengthening Aboriginal health leadership, and investing in co-designed models of care are essential to narrowing the cancer gap and advancing health equity for Aboriginal people in WA and across Australia.

## Figures and Tables

**Table 1:** Study inclusion and exclusion criteria.

Inclusion Criteria	Exclusion Criteria
Western Australia Focus: Studies primarily focusing on cancer-related issues in WA or conducted by researchers based in WA will be included. Inclusion of Aboriginal Peoples: Studies must focus on or include Aboriginal peoples. Publication Standards: Peer-reviewed studies published between 2000 and 2024 will be considered. Comprehensive Cancer Scope: Studies exploring the experiences of Aboriginal individuals with cancer, including prevention, screening, diagnosis, treatment, and end-of-life care, as well as the impact on their families and communities, will be included. Inclusive of all age groups: Studies focusing on cancer across all age groups will be included, ensuring inclusivity and relevance.	Studies that do not focus on Aboriginal people or do not include specific data or insights relevant to Western Australian Aboriginal communities. Studies published before 2000 will be excluded. Studies that do not directly address cancer as part of related lifestyle risks among Aboriginal populations. Non-peer-reviewed studies, conference abstracts, and books.

**Table 2:** Frequency distribution of intervention types.

Intervention Type	Frequency
Community and social	1
Educational	6
Lifestyle	1
Medical	4
Preventive	1
Supportive care	2
Other	2

**Table 3:** Distribution of publications by the age group of the population studied.

Age Group (Years)	Publications
Children (0–18)	8
Young adults (18–24)	13
Adults (24–64)	27
Elderly (65 and over)	20
Not specified	37

**Table 4:** Key recommendations for improving cancer care for Aboriginal populations.

Synthesized Recommendations by Key Areas	Supporting Findings and Recommendations in the Literature
Addressing Barriers and Disparities in Cancer Care Increase awareness and training by providing ongoing education for health professionals on cultural competency and Aboriginal health needs, as well as integrating ALOs into multidisciplinary teams. Improve access by simplifying transport schemes, providing culturally appropriate accommodation, and leveraging telehealth to address barriers posed by remoteness and socioeconomic disparities. Address communication challenges by providing cultural safety training for health service providers and adopting communication styles tailored to Aboriginal patients.	Increase Awareness and Training Durey et al. (2017) [11]: A culturally focused workshop significantly improved healthcare professionals’ confidence in providing culturally safe care to Aboriginal patients, highlighting the need for ongoing professional development. Taylor et al. (2020) [12]: Integrating ALO into multidisciplinary teams improved patient outcomes by facilitating culturally competent care and communication. Shahid et al. (2009) [13]: Employing Aboriginal staff and providing cultural safety training to increase understanding of Aboriginal needs and improve trust between healthcare providers and patients is needed to improve cancer outcomes. Improve Access Shahid et al. (2011) [9]: Identified logistical challenges such as transport and accommodation as significant barriers to cancer care for rural Aboriginal patients. Simplifying transport schemes and offering culturally appropriate accommodation was recommended to address these barriers. Thackrah et al. (2022) [14]: Highlighted the need for the integration of APNs and telehealth services to support remote patients and reduce travel burdens. Taylor et al. (2022) [15]: Telehealth improved accessibility for rural and remote communities by reducing travel requirements while maintaining quality care. Address Communication Challenges Shahid et al. (2013) [8]: Language differences and cultural misunderstandings were major barriers to effective communication. Cultural safety training for healthcare providers and adopting communication styles aligned with Aboriginal patients’ preferences were recommended. Shahid et al. (2009) [13]: Highlighted the importance of building rapport and trust through culturally appropriate communication, emphasizing the need for plain language and interpreters when necessary. Taylor et al. (2021) [16]: ALOs were found to bridge cultural gaps, enhance patient-provider communication, and build trust with Aboriginal patients.
Promoting Effective Education, Communication, and Culturally Appropriate Support Actively involve Aboriginal communities in program design, ensuring they address the unique cultural, social, and practical needs of these populations. Focus on creating culturally safe healthcare environments by incorporating traditional practices and local artwork and fostering mutual trust between Aboriginal patients and healthcare providers. Develop culturally appropriate education resources and health literacy programs to improve cancer awareness and prevention. Expand Aboriginal-led cancer support networks to provide emotional, practical, and cultural support.	Community Involvement Cuesta-Briand et al. (2016) [17]: An Aboriginal women’s cancer support network acted as a cultural broker, creating culturally safe spaces and engaging in grassroots health promotion. This demonstrated the value of actively involving communities in designing and implementing programs. Byers et al. (2018) [18]: Community-led initiatives, co-facilitated by Aboriginal women, effectively increased awareness and access to cancer screening in remote areas. Shahid et al. (2009) [13]: Recommended involving Aboriginal patients and communities in designing healthcare services to address cultural and practical needs and improve participation. Cultural Safety Shahid et al. (2009) [13]: Creating culturally welcoming environments through Aboriginal artwork, traditional practices, and visible Aboriginal staff fostered trust and improved engagement with healthcare services Taylor et al. (2018) [10]: Culturally safe environments, including Aboriginal art, language, and cultural symbols, promoted patient comfort and trust Van Schaik & Thompson (2012) [19]: Advocated for incorporating Aboriginal perspectives and practices into care delivery to improve patient experiences and outcomes. Culturally Appropriate Education Resources Pilkington et al. (2017) [20]: Recommended developing culturally tailored education resources, such as involving Aboriginal breast cancer survivors in messaging to improve awareness and screening participation. Christou et al. (2010) [21]: Identified low awareness of bowel cancer and screening among Aboriginal Australians, emphasizing the need for culturally relevant education materials. Croager et al. (2010) [22]: A culturally tailored cancer education course significantly increased Aboriginal health professionals’ knowledge, underscoring the importance of relevant training materials and approaches. Expand Aboriginal-Led Cancer Support Networks Cuesta-Briand et al. (2015) [23]: Emphasized the need for Aboriginal-led cancer support networks to provide culturally appropriate, emotional, and practical support, vital for addressing the unique challenges faced by Aboriginal cancer patients. Finn et al. (2008) [24]: The Midwest Aboriginal Women’s Cancer Support Group successfully supported Aboriginal women with cancer, bridging gaps in access to care and emotional support, and showcased the importance of expanding similar networks. Thackrah et al. (2022) [14]: Highlighted the potential for APN roles to complement existing support networks by addressing gaps in service delivery and providing culturally safe care.
Enhancing Cancer Screening Participation and Awareness Initiatives Tailor national screening programs to Aboriginal needs by integrating them into primary care and using community-driven approaches. Co-design culturally relevant materials and employ Aboriginal health workers to encourage participation in screenings. Focus on innovative screening techniques (e.g., risk-stratified methods, mammographic density considerations).	Tailor National Screening Programs to Aboriginal Needs Christou et al. (2010) [21]: Highlighted that the National Bowel Cancer Screening Program (NBCSP) does not adequately reach Aboriginal Australians. They recommended integrating screening programs into primary care and using community-driven approaches to improve participation. Taylor et al. (2024) [25]: Argued for co-designed screening initiatives with local communities to address cultural sensitivities and ensure programs meet Aboriginal needs. Shahid et al. (2011) [9]: Found that integrating screening into routine primary care and simplifying access can address logistical and cultural barriers. Co-Design Culturally Relevant Materials and Employ Aboriginal Health Workers Byers et al. (2018) [18]: Demonstrated that educational sessions co-facilitated by Aboriginal women and culturally relevant materials, such as artwork and localized DVDs, increased participation in screening programs. Pilkington et al. (2017) [20]: Recommended involving Aboriginal health workers and breast cancer survivors to design and deliver culturally appropriate education and support for screenings. Cuesta-Briand et al. (2016) [17]: Argued that involving Aboriginal health workers as cultural brokers improved trust and participation in cancer care and screening programs. Focus on Innovative Screening Techniques Carter et al. (2021) [26]: Proposed that risk-stratified screening methods, such as using serum biomarkers for hepatocellular carcinoma, are likely to be cost-effective and beneficial for Aboriginal populations. Darcey et al. (2019) [27]: Identified that mammographic density is a strong predictor of breast cancer risk among Aboriginal women and suggested that innovative techniques tailored to this population could enhance screening accuracy. Taylor et al. (2022) [28]: Recommended adapting screening approaches to local community contexts and exploring new techniques to overcome participation barriers.
Improving Cancer Treatment and Outcomes Implement holistic care approaches, incorporating traditional healing alongside Western treatments. Ensure Aboriginal patients are represented in clinical trials and provided equitable access to advanced treatments. Address late-stage diagnoses by improving early detection and treatment pathways through primary care and community engagement.	Implement Holistic Care Approaches, Incorporating Traditional Healing Shahid et al. (2010) [29]: Some Aboriginal patients use traditional bush medicine and healing practices alongside Western treatments, which enhances their connection to cultural heritage and spirituality. Incorporating traditional healing into care fosters trust and engagement with mainstream healthcare services. Van Schaik & Thompson (2012) [19]: Highlighted the importance of recognizing Aboriginal beliefs about holistic health and integrating these perspectives into care delivery. Tranberg et al. (2016) [30]: Recommended respecting the spiritual and cultural beliefs of Aboriginal patients during treatment planning to enhance trust and adherence to care. Ensure Aboriginal Patients Are Represented in Clinical Trials and Provided Equitable Access to Advanced Jessop et al. (2021) [31]: Identified low participation of Aboriginal children in leukemia clinical trials, which contributed to poorer treatment outcomes. They recommended increasing representation in trials to ensure equitable access to advanced care. Clark et al. (2024) [32]: Stressed the need for Aboriginal Australians to be included in genomics and precision cancer medicine studies as this enhances access to advanced treatments tailored to their specific needs. Condon et al. (2014) [33]: Found that disparities in access to treatment, particularly in rural areas, significantly impact survival rates, highlighting the need for equity in advanced cancer care. Address Late-Stage Diagnoses by Improving Early Detection and Treatment Pathways Shahid et al. (2011) [9]: Identified delayed diagnoses among Aboriginal Australians due to limited awareness, late presentation, and systemic barriers. Emphasized the importance of improving primary care engagement and early detection pathways. Taylor et al. (2024) [25]: Reported that early cancer detection initiatives co-designed with Aboriginal communities increased screening uptake and led to earlier diagnosis. Christou et al. (2010) [21]: Recommended integrating early detection efforts into routine primary care services to reduce the frequency of late-stage cancer diagnoses.
Strengthening Policy and System-Level Intervention Embed Aboriginal health priorities in organizational strategies, guided by frameworks such as the National Aboriginal and Torres Strait Islander Cancer Framework. Improve Aboriginal representation in health governance and develop partnerships with Aboriginal health organizations. Ensure accurate recording of Aboriginal status in healthcare systems for better monitoring and reporting.	Embed Aboriginal Health Priorities in Organizational Strategies Taylor et al. (2022) [15]: Tertiary cancer services implementing the National Aboriginal and Torres Strait Islander Cancer Framework achieved better cultural safety and care outcomes. A whole-of-organization approach ensures sustainable and meaningful inclusion of Aboriginal health priorities. Shahid et al. (2008) [34]: Highlighted that building Aboriginal-specific action plans within cancer organizations fostered a structured and effective approach to meeting Aboriginal health needs. Thompson et al. (2014) [35]: Documented the progress made by state-based Cancer Councils in embedding Aboriginal health priorities, emphasizing the importance of strategic alignment with national frameworks Improve Aboriginal Representation in Health Governance and Partnerships Taylor et al. (2020) [12]: Strong Aboriginal representation in health governance and leadership fosters better alignment with community needs, improving patient outcomes and cultural safety. Thompson et al. (2014) [35]: While some progress has occurred, it was recommended that Cancer Councils recruit more Aboriginal staff, including Board members, and develop stronger partnerships with Aboriginal health organizations to ensure inclusive policy-making. Shahid et al. (2009) [13]: Proposed increasing Aboriginal participation in hospital committees and health governance structures to ensure Aboriginal voices are included in decision-making. Ensure Accurate Recording of Aboriginal Status Condon et al. (2014) [33]: Highlighted that incomplete or inaccurate recording of Aboriginal status in cancer registries results in underreporting of health disparities, making it difficult to design targeted interventions. Luke et al. (2022) [36]: Found significant disparities in referral and access to clinical genetic health services for Aboriginal populations, partly due to inconsistent identification in healthcare systems. Accurate recording would help address these gaps. Shahid et al. (2011) [9]: Recommended improving patient record systems to consistently and accurately capture Aboriginal status as this is critical for effective monitoring and tailored service delivery.
Supporting Families and Communities Throughout their Cancer Journey Provide family-focused support throughout the cancer journey, addressing the financial and emotional burdens they face. Engage communities in health promotion, cancer care design, and end-of-life planning to ensure culturally relevant solutions. Develop culturally safe, home-based care options that involve families in planning and address misconceptions about care.	Provide Family-Focused Support Bell et al. (2021) [1]: Identified that carers of Aboriginal cancer patients often faced financial and emotional challenges. Recommended tailored tools and strategies to address these needs, such as practical support and advocacy for patient care. Thackrah et al. (2022) [14]: Emphasized the importance of family involvement throughout the cancer journey and highlighted the need for emotional and practical support to reduce the burden on family members, especially those who must relocate for treatment. Taylor et al. (2021) [16]: Found that family support is critical for Aboriginal cancer patients but can impose significant burdens on carers. Recommended comprehensive support systems, including financial assistance and emotional support for families. Engage Communities in Cancer Care Design Cuesta-Briand et al. (2016) [17]: Highlighted the role of community engagement in designing culturally relevant cancer support networks that address unique cultural and social needs, such as stigma around cancer and access challenges. Thompson et al. (2019) [37]: Reported that engaging Aboriginal communities in end-of-life planning resulted in culturally respectful solutions and could be assisted by approaches such as sorting cards to facilitate discussions about preferences and addressing burial costs. Shahid et al. (2009) [13]: Community involvement in health promotion and cancer care design improves trust and increases participation in care services. Develop Culturally Safe, Home-Based Care Options Dembinsky (2014) [38]: Highlighted the underutilization of palliative care services among Aboriginal populations due to cultural misconceptions and structural barriers. She recommended strengthening home-based care options that align with cultural practices. Thackrah et al. (2022) [14]: Found Aboriginal people supported the development of culturally safe home-based care models to provide families with more accessible and meaningful care, reducing anxiety and improving outcomes. Shahid et al. (2011) [9]: Recommended expanding home-based services that involve families in planning and address misconceptions about palliative care to improve access and engagement.
Building Research Capacity and Data Collection to Guide Aboriginal and Community-Led Initiatives Conduct further research on Aboriginal cancer outcomes, focusing on disparities in survival rates, treatment access, and cultural influences. Evaluate the effectiveness of culturally tailored interventions and share best practices across services.	Conduct Further Research on Aboriginal Cancer Outcomes Condon et al. (2014) [33]: Highlighted significant disparities in cancer survival rates between Aboriginal and non-Aboriginal Australians, particularly in remote areas. Recommended research to understand the factors contributing to poor outcomes, such as late diagnosis, limited treatment access, and comorbidities. Valery et al. (2013) [39]: Found Aboriginal children had lower five-year survival rates than non-Aboriginal children and argued for further research into patterns of care and compliance to identify areas for improvement. Shahid et al. (2013) [8]: Emphasized the importance of understanding cultural influences on treatment decisions and patient-provider communication to improve engagement and survival outcomes Evaluate the Effectiveness of Culturally Tailored Interventions Durey et al. (2017) [11]: Demonstrated that culturally focused workshops significantly improved healthcare professionals’ ability to provide culturally safe care. Recommended ongoing evaluation of such interventions to ensure sustained impact. Byers et al. (2018) [18]: Reported that community-driven educational initiatives significantly increased cancer screening participation. Suggested sharing these best practices across services to enhance overall effectiveness. Taylor et al. (2018) [10]: Highlighted the lack of information-sharing among cancer services implementing Aboriginal-specific programs. Advocated for evaluating and disseminating successful strategies to foster collaborative learning and improve service delivery.

## Data Availability

All papers are publicly available. The original contributions presented in this study are included in the article/Supplementary Material. Further inquiries can be directed to the corresponding author.

## Supplementary Materials

The following supporting information can be downloaded at: https://www.mdpi.com/article/10.3390/ijerph23060777/s1. File S1. Search Strategy Table. File S2. Qualitative articles. File S3. Quantitative articles. File S4. Review articles. File S5. Mixed methods articles. Refs. [1,2,3,7,8,9,10,11,12,13,14,15,16,17,18,19,20,22,23,24,25,26,27,28,29,30,31,32,33,34,35,36,37,38,39,40,42,43,44,45,46,47,48,49,50,51,52,53,54,55,56,57,58,59,60,61,62,63,64,65,66,67,68,69,70,71,72,73] are cited in Supplementary Materials.

## Abbreviations

The following abbreviations are used in this manuscript:

ALOs	Aboriginal Liaison Officers
APNs	Aboriginal Patient Navigators
WA	Western Australia
WACRH	Western Australian Centre for Rural Health
