# Supplementary material for: A Review of Western Australian Researchers’ Contributions to Understanding Cancer Prevention and Outcomes in Aboriginal People
Source: Int J Environ Res Public Health. 2026 Jun 10;23(6):777. doi: 10.3390/ijerph23060777 (PMC13300084; doi:10.3390/ijerph23060777)
Supplement: Supplementary file 1 [file ijerph-23-00777-s001.zip › Supplementary File S1.pdf]

Supplementary File S1: Search Strategy

Online database search conducted on the 3rd of August, 2024

**Databases**	**Concept 1**	**Concept 2**	**Concept 3**	**Combined terms**	**Total number articles retrieved**
**PubMed Search**	"Australian Aboriginal and Torres Strait Islander Peoples"[mh] OR "Native Hawaiian or Other Pacific Islander"[mh] OR Aboriginal[tiab] OR Aboriginal[tiab] OR "Torres Strait Islander"[tiab] OR "Torres Strait Islanders"[tiab] OR "Aboriginal and Torres Strait Islander "[tiab] OR "First Australians"[tiab] **[59,739]**	Neoplasms[mh] OR cancer[tiab] OR oncology[tiab] OR neoplasia[tiab] OR tumors[tiab] OR tumours[tiab] OR malignancies[tiab] [**4,813,998]**	Western Australia[mh] OR "Western Australia"[tiab] OR "Western Australia"[ad] OR WA[ad] OR Perth[ad] **[215,188]**	Concept 1 + 2+3 **[116]**	Using the filter. Species: Humans Language: English **[107]**

**CINAHL**	Aboriginal OR Aboriginal OR<br>"Torres Strait Islander" OR<br>"Torres Strait Islanders" OR<br>"Aboriginal and Torres Strait<br>Islander " OR "First Australians"<br>[49,543]	Neoplasm* OR cancer* OR oncology OR neoplasia OR tumor* OR tumour* OR<br>malignancies [879,676]	"Western Australia"<br>**[6005**]	Concept 1 + 2+3<br>**[6005**]	**[30]**
**Cochrane Library**	Aboriginal OR Aboriginal OR<br>"Torres Strait Islander" OR<br>"Torres Strait Islanders" OR<br>"Aboriginal and Torres Strait<br>Islander " OR "First Australians"	Neoplasm* OR cancer* OR oncology OR neoplasia OR tumor* OR tumour* OR malignancies	"Western Australia"	Concept 1 + 2+3<br>**[2]**	**[2]**
**Australian**<br>**Aboriginal Health**<br>**Info net**	“cancer”				Filters<br>Type: Journal article,<br>Location: Western<br>Australia
					**[20]**

**Google Scholar**	“Aboriginal” OR “Aboriginal”	“cancer”	“Western Australia”	Concept 1 + 2+3	Filter: Articles published<br><br>2000 onwards<br><br>All articles were added from link 1-link 10 **[193]**
