# Supplementary material for: A Review of Western Australian Researchers’ Contributions to Understanding Cancer Prevention and Outcomes in Aboriginal People
Source: Int J Environ Res Public Health. 2026 Jun 10;23(6):777. doi: 10.3390/ijerph23060777 (PMC13300084; doi:10.3390/ijerph23060777)
Supplement: Supplementary file 1 [file ijerph-23-00777-s001.zip › Supplementary File S2 (QualitativeArticles).pdf]

First Author (Year) Location	Study aim	Study Focus, Design and Population	Findings	Recommendations
Bell et al. (2021) Queensland [1]	Explore the support needs of informal carers of Aboriginal and Torres Strait Islander adults with cancer.	Education, communication, and support<br><br>Interviews, focus groups<br><br>27 participants (15 Indigenous cancer survivors, 12 carers)	Four key themes relating to carers' needs were identified: managing multiple responsibilities, maintaining the carer's own health and wellbeing, accessing practical support and information, and engaging with the health system.<br>Specific needs for carers of Aboriginal and Torres Strait Islander peoples include advocating for the patient, accessing Indigenous support services and health workers, and ensuring the cultural needs of the person are recognised and respected.	Identifying the needs of informal carers of Aboriginal and Torres Strait Islander cancer patients will enable a greater understanding of the support that carers require and inform the development of strategies to meet these needs.<br>Having a tailored assessment tool that captures these areas of need will assist health professionals to systematically identify and address these needs.
Byers et al. (2018) NT [18]	Increase awareness, acceptance of and access to screening mammography among Aboriginal women living in very remote communities in Central Australia.	Screening and prevention<br><br>Qualitative descriptive<br><br>Not specified	A culturally tailored local health promotion initiative significantly increased awareness and access to screening mammography among Aboriginal women in three very remote communities.<br>Educational sessions co-facilitated by senior Aboriginal women and health practitioners effectively raised awareness about breast health, and additional health topics such as cervical screening and nutrition.<br>Culturally relevant materials, such as a locally produced DVD, breast models and artwork were used to encourage participation.	None
Cuesta-Briand et al. (2016) WA [17]	Explore the functions of an Aboriginal women's cancer support network in WA.	Education, communication, and support<br><br>Interviews<br><br>24 participants (3 Aboriginal staff; 8 staff from cancer services; 6 network staff; 4 network clients; 3 potential clients)	The main roles of the Aboriginal women's cancer support network were:<br>Linking and connecting people and services, acting as a "cultural broker" to provide a culturally safe space for Aboriginal women to engage with cancer services and health promotion/screening initiatives.<br>Providing emotional support, though there was tension between Aboriginal stakeholders valuing cultural appropriateness and MSPs prioritizing clinical safety.<br>Engaging in grassroots health promotion and education initiatives to address the "silence about cancer" in the Aboriginal community.<br>Providing practical and financial support.	Ensure adequate ongoing funding and resources to support the sustainability of culturally appropriate Indigenous-led initiatives.<br>Build collaborative relationships between Aboriginal organisations and mainstream cancer service providers<br>Value and honour Aboriginal approaches and expertise.

First Author (Year) Location	Study aim	Study Focus, Design and Population	Findings	Recommendations
Cuesta-Briand et al. (2015) WA [23]	Explore different understandings of how an Indigenous women's cancer support group should operate and the impact of unresolved tensions between Indigenous and mainstream healthcare perspectives.	Education, communication, and support<br><br>Interviews<br><br>24 participants (3 Aboriginal staff; 8 staff from cancer services; 6 network staff; 4 network clients; 3 potential clients)	Unresolved tensions between Indigenous and non-Indigenous participants regarding the need for structure versus flexibility in the operations of an Indigenous cancer support network negatively impacted the working relationship and threatened the network's sustainability.<br>Indigenous participants preferred a flexible, organic approach, while mainstream service providers believed a more structured approach was needed for sustainability.	Address and resolve tensions between different perspectives on service delivery through two-way learning and building trust and respect.<br>Provide formal training and mentoring relationships for the network. Recognize the important role of volunteers in bridging community and health services.<br>Provide greater support for Aboriginal leadership in cancer care.
Dembinsky (2017) WA [52]	Explore the ways in which breast cancer health information is socially circulated and consumed among the Yamatji community in WA.	Education, communication, and support<br><br>Qualitative ethnographic design<br><br>Not specified	Yamatji women actively negotiate and shape the health information they consume, rather than passively accepting it.<br>For effective health information dissemination, providers and consumers need to collaborate.<br>Verbal communication following cultural protocols (yarning) is the most trusted form of communication, while written and audiovisual materials are less accepted due to issues of trust and cultural appropriateness.	None
Dembinsky (2014) WA [38]	Explore the perceptions and use of palliative care services among the Yamatji people, particularly focusing on individuals affected by breast cancer.	Barriers and disparities<br><br>Interviews<br><br>28 participants (8 health professionals; 10 Aboriginal women with cancer; 10 family members)	Yamatji women with breast cancer underutilize palliative care services due to misperceptions about what palliative care entails, cultural views on death, and structural barriers to accessing services.<br>Education and outreach programs alone are not sufficient to increase Yamatji use of palliative care services, as they do not address the cultural and structural barriers.	Expand and strengthen home-based palliative care services in Yamatji country, while considering cultural barriers<br>Develop education and outreach programs to address misperceptions about palliative care.<br>Further research into Aboriginal perceptions of palliative care, particularly evaluating educational interventions and home-based palliative care services.
Finn et al. (2008) WA [24]	Explore the effectiveness of the Midwest Indigenous Women's Cancer Support Group (IWCSG) to support Indigenous women with cancer, along with their caregivers and relatives.	Education, communication, and support<br><br>Interviews and observation<br><br>22 participants (11 health service providers, the IWCSG coordinator, 10 women who had been linked to IWCSG support)	The IWCSG provides emotional and practical support to Indigenous women with cancer and their carers/relatives, helping them overcome barriers to accessing cancer screening and treatment services.<br>The IWCSG acts as a "cultural bridge" between Indigenous people and non-Aboriginal health service providers, improving communication and understanding.<br>The IWCSG has the potential to serve as a model for support groups for the Indigenous population for other serious chronic diseases, both in metropolitan and regional areas.	Secure ongoing funding and a full-time coordinator for the IWCSG to expand and develop its operations.<br>Develop systems to better track and evaluate the services and outcomes of the IWCSG.<br>Conduct action research to facilitate effective collaborative partnerships between the IWCSG and mainstream health service providers.<br>Use the lessons learned from the IWCSG to inform a model for establishing similar support groups in other areas and for other health issues.

First Author (Year) Location	Study aim	Study Focus, Design and Population	Findings	Recommendations
Haigh et al. (2016) WA [40]	Evaluate the implementation and usage of a DVD developed to educate Aboriginal people in WA about bowel cancer and the importance of screening.	Screening and prevention<br><br>Interviews, focus groups<br><br>67 participants (individuals involved in the development of the DVD, health professionals, and Aboriginal community members)	The DVD developed to promote bowel cancer screening among Aboriginal Australians was well-received and considered a suitable educational resource. However, the DVD was not widely distributed or utilized, similar to a previous flipchart resource.<br>The underutilization of the DVD was attributed to the lack of engagement of health service providers (HSPs) with the National Bowel Cancer Screening Program, which sends screening kits directly to participants without involving HSPs.	Improve distribution and follow-up strategies for health promotion resources like the DVD.<br>Increase involvement of primary care services in the distribution and follow-up of FOBT kits for the NBCSP.<br>Provide training and clearly articulate the role of health service providers, especially nurses, in promoting the NBCSP
Lyford et al. (2018) WA [62]	Explore the underrepresentation of Aboriginal cancer patients in a newly established regional radiotherapy service in South West WA.	Barriers and disparities<br><br>Interviews<br><br>24 participants (21 service providers, 3 Aboriginal patients)	Aboriginal cancer patients who received treatment at the regional radiotherapy service were satisfied with the care and support they received, despite the complex challenges they faced in accessing and continuing treatment.<br>Service providers suggested several reasons for the low numbers of Aboriginal patients presenting at the radiotherapy service, including late cancer diagnosis, perceptions of cultural insensitivity, financial barriers, and issues with accurately recording patients' Aboriginal status.	Provide a supportive environment that addresses the access challenges faced by rural Aboriginal cancer patients from initial diagnosis through to follow-up care.<br>Educate Aboriginal people about cancer symptoms and the importance of seeking timely medical advice.<br>Establish enhanced discharge planning and follow-up support arrangements to address the ongoing needs of rural Aboriginal cancer patients.<br>Improve the recording of Aboriginal status in patient data.<br>Ensure that all healthcare staff understand their responsibility in providing culturally appropriate treatment and are trained in the appropriate collection and recording of Aboriginal status.

First Author (Year) Location	Study aim	Study Focus, Design and Population	Findings	Recommendations
Pilkington et al. (2017) WA [20]	Examine perspectives on breast screening among Aboriginal women in WA including the factors that impact participation in the BreastScreen WA screening mammogram program.	Screening and prevention<br><br>Interviews, focus groups, yarning sessions<br><br>65 participants (Aboriginal consumers and Aboriginal health professionals)	Aboriginal women in the study were generally willing to have a mammogram.<br>Motivating factors were having a genetic predisposition, a desire to maintain their health and set an example.<br>Key barriers to screening participation included lack of understanding about the screening process, inadequacies in cultural appropriateness in the screening program, cultural beliefs about cancer, and competing life demands.<br>Enablers to screening participation included culturally appropriate education and support from other Aboriginal women in the community.	Logistical recommendations:<br>Cater for last minute appointments and "drop in" sessions to increase flexibility.<br>Allocate block booking times for Aboriginal women to create a supportive environment.<br>Extend transport strategies to include metropolitan and regional areas.<br>Cultural recommendations:<br>Provide cultural competence training for staff.<br>Increase the number of Aboriginal staff at screening facilities.<br>Involve respected Aboriginal women in the screening process to provide support.<br>Educational recommendations:<br>Increase the number of Aboriginal educators providing information about screening.<br>Include Aboriginal breast cancer survivors in support and education messaging.<br>Develop resources to encourage Aboriginal women to participate in screening.
Schofield et al. (2011) WA [67]	Identify the challenges faced in diagnosing and managing Lynch Syndrome (LS) within an Indigenous family living in a remote West Australian community, particularly regarding access to genetic counselling and testing in rural areas.	Barriers and disparities<br><br>Retrospective case study<br><br>Aboriginal cancer patient and extended family	Routine screening of CRC specimens using MSI and IHC testing led to the identification of an Indigenous family with Lynch syndrome that had not been previously recognized based on clinical criteria alone.<br>Managing the Indigenous family with Lynch syndrome from a remote location highlighted challenges in providing culturally appropriate clinical genetic services.<br>Flexibility in standard clinical genetic protocols and the involvement of a local GP were key to the successful management of this family.<br>Family members gained a better understanding of their genetic risks, resulting in increased participation in genetic testing; seven out of eight siblings opted for testing, with four confirmed as mutation carriers.	None

First Author (Year) Location	Study aim	Study Focus, Design and Population	Findings	Recommendations
Shahid et al. (2016) WA [7]	Examine the factors contributing to the delayed diagnosis of cancer among Aboriginal Australians from both patient and health service providers' perspectives.	Barriers and disparities<br><br>Interviews<br><br>92 participants (30 Aboriginal cancer patients and family members, 62 health service providers)	Multiple factors at the contextual, health system, and patient levels contribute to delayed cancer diagnosis among Aboriginal Australians.<br>Contextual factors included the intergenerational impact of colonization, racism, and socioeconomic deprivation, which negatively affected trust in healthcare professionals.<br>Health service-related factors included limited access to medical services, long waiting periods for appointments and diagnostic tests, and a shortage of culturally competent health professionals.<br>Patient appraisal of symptoms and decision-making were significant, as many patients experienced fear, denial, and embarrassment regarding their symptoms, leading to procrastination in seeking help.	Focus on the primary care sector and encourage GPs to be more proactive in investigating potential cancer symptoms and facilitating timely referrals for further investigation.<br>Build trust and providing cultural safety training for healthcare service providers to ensure a culturally safe environment and improve communication and relationships between Aboriginal patients and non-Aboriginal healthcare providers.<br>Implement community-based cancer awareness programs to educate Aboriginal people about the importance of early diagnosis and treatment, aiming to dispel fears associated with cancer.<br>Use patient navigators and telehealth services to address barriers related to remoteness and lack of access to healthcare services.
Shahid et al. (2013) WA [8]	Explore the perspectives of cancer service providers (CSPs) regarding their experiences working with Aboriginal patients and their families who are dealing with cancer and identify barriers that impede effective communication between CSPs and Aboriginal patients.	Barriers and disparities<br><br>Interviews<br><br>62 participants (Aboriginal and non-Aboriginal cancer service providers)	CSPs' lack of knowledge about the needs of Aboriginal people with cancer and Aboriginal patients' limited understanding of the Western medical system were major barriers to communication between CSPs and Aboriginal patients.<br>Communication was impeded by language differences, differing communication styles, and differing concepts of time, which created challenges for timely appointments and adherence to treatment plans.<br>Marginalization of Aboriginal people within mainstream society and Aboriginal people's distrust of the healthcare system also impeded communication.<br>Potential solutions include recruiting more Aboriginal staff, providing cultural training, and improving coordination and continuity of care.	Ensure cultural competency at the clinical, organizational, and system level by recruiting and training Aboriginal staff, providing cultural safety training to all staff, and improving patient record systems to better identify Aboriginal patients.<br>Make the service environment more welcoming to Aboriginal people by consulting with local Aboriginal communities, displaying inclusive artwork and imagery, and having visible Aboriginal staff.<br>Develop appropriate cancer education for Aboriginal people and evaluate impact.<br>Adopt a holistic, patient-centred approach that takes the time to understand and address the patient's cultural needs and social context, rather than just focusing on the most effective clinical treatment.<br>Improve the coordination of care between cancer treatment services, primary care and community-based care.<br>Use interpreters as needed and use plain language to communicate with Aboriginal patients.

First Author (Year) Location	Study aim	Study Focus, Design and Population	Findings	Recommendations
Shahid et al. (2011) WA [9]	Investigate the experiences and barriers faced by Aboriginal people in accessing cancer services and treatment in WA.	Barriers and disparities<br><br>Interviews<br><br>30 participants (14 adult cancer patients and survivors, 16 family members)	A need for practical and emotional support in all aspects of the cancer journey was identified, particular for rural and remote patients.<br>Infrastructure and logistical problems that impeded Aboriginal patients' access to cancer treatment included transport, accommodation, travel and service expenses, concerns about the hospital environment, displacement from family and lack of appropriate support persons.	System-level changes are needed to ensure cultural safety, social support, and better coordination between cancer treatment services and primary healthcare to improve outcomes for Aboriginal people with cancer.<br>Transport: simplify the Patient Assisted Travel Scheme (PATS) process and expand outreach delivery of specialist care, such as telehealth and community-based services.<br>Accommodation: provide culturally safe, accessible facilities with room for family members to stay.<br>Hospital environment: make health services more welcoming (Aboriginal artwork, traditional foods, outdoor access) and ensure access to Aboriginal interpreters.<br>Employ Aboriginal Liaison Officers (ALOs) and patient advocates to provide practical and emotional support.<br>Improve coordination between hospitals and primary care services to ensure continuity of care and follow-up support.
Shahid et al. (2010) WA [29]	Explore the beliefs, understanding, and experiences of Aboriginal Australians on the use of bush medicine and traditional healing practices for cancer treatment.	Treatment and outcomes<br><br>Interviews<br><br>11 participants (cancer patients, family members of people who had died from cancer, and Aboriginal health service providers)	Some Aboriginal Australians use traditional bush medicine and healing practices as part of their cancer treatment, as it helps them connect with their cultural heritage and spirituality.<br>Spiritual beliefs and a holistic view of health are important factors that influence some Aboriginal patients' choices in cancer treatment, including the use of traditional medicine.<br>Healthcare providers need to recognize and understand Aboriginal knowledge and worldviews, and accept that traditional healing can be an important complement to Western medical treatments.	Health service providers should recognize and understand the use of traditional Aboriginal medicine and healing, as this can improve Aboriginal people's access and engagement with mainstream health services.<br>Further research is needed to understand the therapeutic value of traditional Aboriginal medicine, so that it can be better integrated with Western medical approaches.

First Author (Year) Location	Study aim	Study Focus, Design and Population	Findings	Recommendations
Shahid et al. (2009) WA [13]	Explore Aboriginal cancer patients' views about effective communication between Aboriginal people and health service providers within hospital settings in WA.	Barriers and disparities<br><br>Interviews<br><br>30 participants (14 Aboriginal cancer patients, 16 family members)	Communication issues and lack of cultural understanding between Aboriginal patients and healthcare providers were major barriers to effective cancer care.<br>Key issues impairing communication between Aboriginal patients and healthcare providers included: fear of the medical system and of being disempowered; mistrust; collective memories of the experience of colonisation and its aftermath; lack of understanding of Aboriginal customs, values, lifestyle and the importance of family and land; and experiences of racism.<br>Impediments to communication included language barriers, non-verbal communication issues, inadequate information provided by healthcare professionals, and difficulties establishing ongoing personal relationships.<br>Barriers contributed to a loss of trust among Aboriginal patients, impacting their willingness to engage in or continue treatment.	Recruit Aboriginal people onto hospital committees and increase the number of Aboriginal staff.<br>Make the hospital environment more welcoming and use interpreters or relevant support people.<br>Allow time to build rapport and establish trust with patients.<br>Provide cultural safety training for all hospital staff and improve communication skills through targeted training.<br>Provide care and follow-up services closer to patients' homes when possible and strengthen the links between the hospital, primary care community-based support.<br>Use plain language when communicating with Aboriginal patients and their families.<br>Ensure that Aboriginal patients and their families fully understand the prescribed treatment, follow-up, and management plans.
Shahid et al. (2009) WA [68]	Explore the perceptions, beliefs and understanding of cancer of Aboriginal people living in WA.	Barriers and disparities<br><br>Interviews<br><br>37 participants (14 adult cancer patients and survivors, 16 family members, 7 Aboriginal health service providers)	Aboriginal people's beliefs about cancer impacted their decisions to access cancer services.<br>Participants expressed fear and fatalistic views about cancer, often associating it with death, which contributed to delayed diagnosis and treatment.<br>Limited knowledge about cancer, its symptoms, and treatment options were common, leading to low participation in screening programs.<br>Cultural factors, including beliefs about spirituality, shame, and the stigma associated with cancer, as well as a preference for traditional healing methods, were influential.<br>Distrust of the healthcare system was noted as a barrier to seeking and adhering to treatment.	Address Aboriginal peoples' beliefs about cancer when developing educational, screening and treatment approaches.<br>Ensure cancer services and information are culturally appropriate for Indigenous people.<br>Health care providers need to understand and appreciate cultural differences in beliefs and attitudes towards cancer to provide appropriate care and encourage early detection and treatment.<br>Reduce barriers to access, increase visibility of Indigenous cancer survivors, and focus on early diagnosis to improve cancer outcomes in Indigenous communities.

First Author (Year) Location	Study aim	Study Focus, Design and Population	Findings	Recommendations
Shahid et al. (2009) WA [69]	Explore methodological considerations in conducting research into the beliefs, experiences, and perspectives of Aboriginal Australians with cancer.	Barriers and disparities<br><br>Interviews<br><br>50 participants (30 Aboriginal people affected by cancer, 20 health service providers)	The research process needs to be relationship-based, respectful, culturally appropriate and inclusive of Aboriginal people.<br>Researchers are accountable to participants and the wider community for reporting findings and translating the research to benefit the Aboriginal community.<br>The researchers supported the establishment of an Indigenous Women's Cancer support group as an outcome of the research.	Prioritize responsible, socially just research that builds capacity, develops positive relationships, and amplifies Indigenous voices.<br>Collaborate with Indigenous researchers as part of decolonizing research methodologies and incorporating culturally appropriate processes.<br>Be attentive to the culture and traditions of the population, make the research process participatory and inclusive of the community, and provide feedback to the community.
Shahid et al. (2008) Australia [34]	Conduct an environmental scan within state-based Cancer Councils in Australia to map activities related to cancer service provision focusing on Indigenous Australians.	Education, communication, and support<br><br>Environmental scan, interviews<br><br>Staff from the Cancer Councils of the ACT, Tasmania, Victoria, NSW, SA and WA.	Most Cancer Councils have tried to work with Indigenous communities on cancer issues, however there were difficulties in building and sustaining relationships with Indigenous organisations.<br>Lack of dedicated staff time for Indigenous issues and lack of Indigenous staff impeded progress, with only one Cancer Council having an Indigenous staff member at the time of the project.<br>Lack of Indigenous-specific resources was a barrier to cancer education, but some Cancer Councils have developed such resources.	Cancer Councils need to build and improve regional or local partnerships with Indigenous organisations and communities.<br>Cancer Councils should continue to build capacity around cancer within the Indigenous health sector, including through training and networking opportunities.<br>Cancer Councils should provide cultural awareness training for their own staff to better understand and serve Indigenous communities.<br>Involvement of Indigenous people should be a crucial factor in all aspects of cancer-related service delivery.
Taylor et al. (2024) NT [25]	Analyse HP perspectives and approaches to the provision of screening for Aboriginal Australians in the NT.	Screening and prevention<br><br>Interviews<br><br>50 staff from 15 health services	Cancer screening provision in remote and very remote primary health care clinics in the NT is variable, with some clinics seeing it as a major gap and others not prioritizing it due to lack of resources and high burden of other conditions. However, some clinics see screening as an area where they are performing well, with systematic screening, targeted programs, and high screening rates.<br>There was a range of perceptions about the breast and cervical screening programs, but participants unanimously reported that the bowel screening kit was not culturally appropriate and led to low uptake.	Fund and support NT cancer screening services to utilize co-design approaches with local consumers and service providers to improve how cancer screening programs are delivered.<br>Adopt a more holistic approach to screening by removing the siloed approach in national screening programs.<br>Offer cancer screening to patients who have travelled to a regional centre for a medical appointment.<br>Increase funding, resourcing and staffing of remote and very remote primary health care (PHC) clinics to enable a greater focus on cancer screening.<br>Develop education for PHC staff to cover incidence of cancer, the importance of cancer screening and how to engage Aboriginal patients with screening.<br>Develop strategies to recruit and retain the PHC and screening workforce, including increasing the number of Aboriginal health professionals.

First Author (Year) Location	Study aim	Study Focus, Design and Population	Findings	Recommendations
Taylor et al. (2022) Queensland, Victoria [15]	Explore how three Australian cancer services performed when assessed against two national best practice guidelines: the National Aboriginal and Torres Strait Islander Cancer Framework (Cancer Framework) and the National Safety and Quality Health Service (NSQHS) User Guide for Aboriginal and Torres Strait Islander Health (User Guide).	Education, communication, and support<br><br>Interviews<br><br>43 participants (35 hospital staff, 5 Indigenous people with cancer and 3 family members)	Although all three services had previously been identified as "high performing" in their care for Indigenous cancer patients, only two services performed well when compared to the User Guide, showing that sustaining high performance this space can be challenging.<br>All three tertiary cancer services struggled with the upstream priorities of the Cancer Framework, such as education, prevention, screening, and early diagnosis.<br>The importance of organizational commitment, leadership, and the need for a whole-of-organization approach to embed and sustain culturally safe practices<br>Services that have successfully implemented the national User Guide are in a stronger position to implement the Cancer Framework and Optimal Care Pathway for Aboriginal and Torres Strait Islander People with Cancer (OCP).	Health services aiming to improve outcomes for Indigenous cancer patients should adopt a whole-of-organization approach to implement and sustain culturally safe practices.<br>Embed the six actions outlined in the NSQHS User Guide as a foundation for broader improvements.<br>The treatment-focused OCP may be a more appropriate framework for measuring the performance of tertiary services than the Cancer Framework.<br>Ensure long-term, sustainable change through prioritizing Indigenous health, involving Indigenous staff, and ongoing monitoring.<br>Form partnerships with Cancer Councils, Indigenous health organizations, and primary care providers to enhance the delivery, reach, and cultural safety of services for Indigenous cancer patients.<br>Indigenous-led research is needed to identify and evaluate successful cancer delivery programs for Indigenous Australians.
Taylor et al. (2021) Queensland, Victoria [16]	Explore the experiences of Indigenous cancer patients and their families within two high-performing cancer services and determine how these experiences aligned with the strategies implemented by the services to improve care.	Education, communication, and support<br><br>Interviews<br><br>31 participants (23 hospital staff, 5 Indigenous people with cancer and 3 family members)	Indigenous cancer patients and their families reported positive experiences while receiving treatment at the two cancer services, and emphasised the importance of support, good communication, and involvement of Indigenous Liaison Officers (ILOs).<br>ILOs supported patients and their families, facilitated communication, and bridged cultural gaps.<br>The majority of participants reported challenges navigating the health system in the time between first experiencing systems and reaching the cancer service, with delays, misdiagnosis, and poor communication from health providers all reported as barriers.<br>Family support was crucial for Indigenous cancer patients, but also placed a significant burden on family members, especially those who had to relocate.	Health services must prioritize and make an ongoing commitment to strengthen the cultural safety of their services.<br>Documents such as the OCP and NSQHS User Guide provide important guidance on this.<br>Increase the number of ILOs employed in health services and assigned to cancer services.<br>Need for more comprehensive coordination between primary and tertiary care providers.<br>Increase the use of telehealth for pre-admission consultations and pre-hospital orientation, particularly for regional and remote patients.<br>Family involvement should be offered to the patient throughout the cancer journey and family should be supported.<br>Further research is needed to identify and evaluate successful cancer service delivery initiatives for Indigenous Australians.
Taylor et al. (2020)	Investigate how effective support and strategies can build a	Education, communication, and support	Two health services demonstrated positive support for their Indigenous workforce through: strong executive leadership, proactive employment strategies, the	Committed health service leadership with Indigenous health as a core value within the organization's mission and strategic plans.

First Author (Year) Location	Study aim	Study Focus, Design and Population	Findings	Recommendations
Queensland, Victoria [12]	robust Indigenous health workforce within mainstream health services, specifically focusing on two Australian cancer services.	Interviews<br><br>32 participants (24 hospital staff, 5 Indigenous people with cancer and 3 family members)	presence of an Indigenous Health Unit, clearly defined roles for ILOs, inclusion of ILOs within multidisciplinary teams, opportunities for professional development, a supportive work environment, and a culture of respect.<br>Positive outcomes from having a strong Indigenous workforce included: improved patient outcomes, increased patient engagement, better adherence to treatment, increased recruitment and retention of Indigenous staff and improved job satisfaction.	Develop clear and proactive employment strategies to set measurable targets for recruiting and retaining Indigenous staff.<br>Increase engagement with existing Indigenous staff, patients and local communities and seek input on Indigenous health.<br>Create a welcoming work environment through display of local Indigenous artwork and flags in all departments.<br>Assess recruitment processes and remove barriers for Indigenous job-seekers.<br>Establish an Indigenous employee network or mentoring program for Indigenous staff.<br>Ensure all staff complete regular, mandatory health-focused cultural awareness training.<br>Implement systems to ensure ILOs are notified of new Indigenous patients and include ILOs in multidisciplinary teams to advocate for patients and facilitate culturally competent care.
Taylor et al. (2018) Australia [10]	Identify and describe the Indigenous-specific programs and initiatives implemented by cancer services across Australia that aimed to meet the needs of Indigenous cancer patients and their families.	Education, communication, and support<br><br>Interviews<br><br>20 staff from 14 cancer services	Many Indigenous-specific programs and initiatives are being implemented by cancer services across Australia to provide culturally appropriate care, but details of these initiatives are not widely known.<br>Key activities included employing Indigenous staff, providing cultural awareness training, incorporating flexible clinical practices, and making changes to the physical environment to ensure cultural safety.<br>Services highlighted the importance of cross-cultural communication, involvement of Indigenous health workers, and family engagement throughout the treatment process.<br>There was variation between services, with some being more advanced in their efforts.<br>Challenges included a lack of awareness among service providers regarding what other services were doing and limited opportunities for information sharing, particularly in regional and remote areas.	Need for enhanced collaboration, networking, and partnerships among cancer services, particularly between Indigenous and mainstream health service providers, to share successful strategies and initiatives.<br>Further research to evaluate these programs and initiatives and showcase the more effective approaches to Indigenous cancer care.<br>Additional recommendations for cancer services included: provide ongoing cultural awareness training for all staff, employ more Indigenous staff, create welcoming and culturally safe environments by incorporating local artwork and language and expand outreach services and telemedicine improve access for Indigenous patients in remote and regional areas.

First Author (Year) Location	Study aim	Study Focus, Design and Population	Findings	Recommendations
Thackrah et al. (2022) WA [14]	Explore the experiences of Aboriginal cancer patients and carers regarding their cancer journeys, with the aim of informing the development of an Aboriginal Patient Navigator (APN) model for the WA healthcare system.	Education, communication, and support<br><br>Qualitative research<br><br>16 participants (11 cancer patients, 5 carers of family members or friends with cancer)	Patients and carers identified numerous gaps and challenges in cancer care delivery, including lack of accommodation, financial burdens, travel, being "off-Country", and miscommunication with health professionals.<br>Family and cultural connectedness were key sources of support.<br>All participants supported an APN role to address gaps in service delivery and provide culturally safe support, which could increase access, reduce anxiety, and improve outcomes.	Implement an APN role in the WA healthcare system to support Aboriginal cancer patients and their families.<br>The APN role should be clearly defined and designed to complement the work of existing Aboriginal Health Liaison Officers (AHLOs) and be integrated into the healthcare team.<br>The APN role should include both male and female positions to accommodate cultural sensitivities.<br>The APN role should be funded as a long-term commitment.<br>A pilot program in one hospital with evaluation could assess the APN's effectiveness and inform wider implementation of the role.
Thompson et al. (2019) WA [37]	Understand Aboriginal people's perspectives and experiences related to end-of-life wishes and decision-making	Education, communication, and support<br><br>Facilitated group discussion, interviews<br><br>Group discussions with Aboriginal community members and health service providers, interviews with 10 Aboriginal participants (8 people with a cancer diagnosis, 2 Aboriginal cancer support group staff)	Aboriginal people were willing to engage in discussions about end-of-life wishes when provided with a safe and culturally appropriate environment.<br>Key issues included concerns about wills, preferences regarding burial versus cremation, and the high costs associated with funerals.<br>Cancer patients emphasized the importance of family involvement in end-of-life decision-making and highlighted the potential for family conflict if wishes were not clearly communicated.<br>There were mixed perceptions of palliative care, with some participants associating it with imminent death, while others held it in high regard.<br>Participants expressed interest in public celebrations of life, using sorting cards to clarify end-of-life wishes, and community-based solutions like coffin-making to manage funeral expenses.	Providing opportunities for Aboriginal people to discuss grief and loss around end-of-life issues can be beneficial.<br>Resources like the Palliative Care Australia's Dying to Talk materials can be useful to facilitate discussions about end-of-life preferences among Aboriginal communities.<br>Sorting cards, facilitated by experienced facilitators, can be an effective way to encourage discussions about end-of-life wishes among Aboriginal people.<br>Community-led initiatives, such as crafting personalized coffins or creating memorial spaces, could help make funeral arrangements more meaningful and cost-effective.

First Author (Year) Location	Study aim	Study Focus, Design and Population	Findings	Recommendations
Thompson et al. (2014) Australia [35]	Report the findings of an environmental scan conducted in 2010 across all State and Territory Cancer Councils in Australia. The scan aimed to document progress, share lessons learned, and assess the range of activities and initiatives undertaken to support Indigenous cancer control.	Education, communication, and support<br><br>Environmental scan through interviews and data analysis<br><br>19 participants (Cancer Council staff from all Cancer Councils in Australia)	Since the 2006 review most Cancer Councils had increased activities aimed at improving Indigenous cancer outcomes, though Indigenous staff numbers remained low, and no Cancer Council had an Indigenous Board member.<br>There was evidence of enhanced partnerships with Indigenous organizations and increased acknowledgment of Indigenous issues in policy documents.<br>Some Councils had developed culturally relevant resources and training programs.<br>Challenges such as sustaining relationships, resource limitations, and the need for greater cultural competence persisted.<br>The establishment of the national Aboriginal and Torres Strait Islander Subcommittee of the Cancer Council Australia enhanced opportunities for sharing information and national coordination of activity.	Cancer Councils should continue to recruit and support Indigenous staff through peer mentorship programs and ensure staff cultural competence through training.<br>Develop Indigenous-specific action plans, strategic engagement with Indigenous communities, and increased representation on Boards and committees.<br>Enhance visibility and access to Cancer Council services within Indigenous communities through targeted marketing and culturally relevant resources<br>Strengthen partnerships with Aboriginal Community Controlled Health Services.<br>Develop a national repository of cancer resources for Indigenous Australians.<br>Identify barriers and facilitators for Indigenous engagement in cancer screening and care, integrate Indigenous health issues into programs, and encourage research on innovative service delivery approaches.
Thompson et al. (2011) WA [73]	Identify Aboriginal people's views regarding the factors influencing their participation in cancer care, including their beliefs about cancer and experiences with cancer services.	Barriers and disparities<br><br>Interviews<br><br>30 participants (Aboriginal people affected by cancer)	Relationships and respect shown by hospital staff were the most important factors for Aboriginal participants.<br>Key areas of concern about the hospital environment emerged: being alone and lost in a large system; poor communication; practical issues such as costs, transportation, and family responsibilities; importance of Aboriginal support persons; and need for connection to community.<br>While the design of the hospital environment was important, it was secondary to the necessity of building trust and fostering culturally safe interactions.	Recommendations for physical design of hospitals:<br>Locate an Aboriginal Welcome Desk at the hospital entrance staffed by Aboriginal people.<br>Create a welcoming physical environment using colour, texture, light, and cultural symbolism.<br>Build hospital rooms large enough to accommodate extended Aboriginal families.<br>Provide affordable childcare and consider privacy/gender needs.<br>Ensure there is safe, low cost accommodation for patients and family members.<br>Ensure access to information and resources to improve cross-cultural understanding.
