# Supplementary material for: A Review of Western Australian Researchers’ Contributions to Understanding Cancer Prevention and Outcomes in Aboriginal People
Source: Int J Environ Res Public Health. 2026 Jun 10;23(6):777. doi: 10.3390/ijerph23060777 (PMC13300084; doi:10.3390/ijerph23060777)
Supplement: Supplementary file 1 [file ijerph-23-00777-s001.zip › Supplementary File S3 (QuantitativeArticles).pdf]

First Author (Year) Location	Study aim	Study Focus, Design and Population	Findings	Recommendations
Abdalla et al. (2023) WA [43]	Examine hospitalization trends for physical diseases and estimated the associated inpatient care costs in all 5-year childhood cancer survivors (CCS) diagnosed in WA from 1982 to 2014.	Incidence and mortality<br><br>Retrospective cohort study<br><br>2,938 CCS and 24,972 comparisons (138 Indigenous CCS and 1,523 comparisons)	All-cause hospitalization in CCS resulted in greater expenditures on inpatient services compared with the general population.<br>Higher hospitalization rates were associated with female gender, diagnosis with bone tumours, cancer diagnosis age between 5 and 9 years, multiple childhood cancer diagnoses, multiple comorbidities, higher deprivation, increased remoteness, and Indigenous status.	Long-term follow-up healthcare services to prevent disease progression and mitigate the burden of physical morbidity on CCS and hospital services.<br>Prioritize the delivery of targeted and integrated healthcare services, as this can effectively alleviate the burden of hospitalization on survivors, their families, and the healthcare system.
Banham et al. (2023) NSW [2]	Examine the potential for the mitigating effects of more frequent contact with GPs in ensuring local-stage at diagnosis and to understand the characteristics of Aboriginal people diagnosed with cancer including their experience with comorbidity, use of GP services, and any influence of GP use on stage of cancer at diagnosis.	Barriers and disparities<br><br>Retrospective cohort study<br><br>4,084 Aboriginal and 249,037 non-Aboriginal residents of New South Wales aged 50 years or older who were first diagnosed with cancer between 2003 and 2016	Comorbidity, along with age and socio-economic disadvantage, has a negative effect on detecting cancer at a localised stage, and high GP contact partially offsets that effect. The mediating effect of high GP contact could arise in several ways the first of which is by encouraging participation in cancer screening.<br>Older Aboriginal Australians diagnosed with cancer experience more comorbid conditions and more socioeconomic disadvantage than other Australians, which are negatively related to earlier cancer diagnosis.	More frequent GP contact may lead to earlier detection of cancer among the Aboriginal population of NSW.
Bernardes et al. (2019) WA, NSW, NT, Victoria [45]	Use a culturally specific tool to assess the supportive care needs of Indigenous Australian cancer patients and describe the prevalence of unmet need across the participating jurisdictions.	Treatment and outcomes<br><br>Cross-sectional study<br><br>145 Indigenous adults diagnosed with cancer.	Most Indigenous Australians living with cancer experience unmet supportive care needs. Physical/psychological and practical/cultural concerns were identified as priority areas for Indigenous cancer patients. Moderate to high unmet needs most commonly occurred in physical/psychological (46%) and practical/cultural (34%) domains. 'Money worries' was the most frequently reported unmet need (20%).	Develop targeted approaches for optimal care including:<br>Routine screening of unmet needs for Indigenous cancer patients using culturally appropriate tools<br>Facilitating access to cancer care for rural and remote communities through tele-oncology models<br>Providing informative discharge summaries for continuity of care at the community level<br>Implementing an optimal care pathway for Aboriginal and Torres Strait Islander people with cancer to assist health professionals in providing patient-centred care, including assessing and addressing supportive care needs

First Author (Year) Location	Study aim	Study Focus, Design and Population	Findings	Recommendations
Bernardes et al. (2021) Queensland [44]	Improve our understanding of Indigenous patients' experience and the quality of end-of-life care by characterizing the hospital admissions and identifying priority needs of Indigenous Australians with cancer during the last year of lives.	Treatment and outcomes<br><br>Cross-sectional study<br><br>58 Indigenous cancer patients	While most of the Indigenous patients with cancer died in hospital (78%), only a small proportion received palliative care (15%).<br>All patients had at least one hospital admission within the last year of their life.<br>Just under half (48%) did not report any unmet needs.<br>Worry about treatment results, money worries and anxiety were the most frequently reported unmet needs.	Increasing the awareness about the value of palliative care in symptom management and quality of life to patients and carers.<br>Qualitative studies to explore worry in the palliative setting to better understand patients' perspectives and develop and implement strategies to address these worries.<br>Earlier initiation of community-based palliative care to reduce the number of unplanned admissions, reduce the cost of in-hospital end-of-life-care and respect patient preferences.
Bilkey et al. (2021) WA [46]	Provide an overview of the rare cancer landscape in WA and support researchers, clinicians and policy makers to better understand areas of need and how to best support Western Australians living with rare cancers.	Incidence and mortality<br><br>Descriptive epidemiology: analysis of cancer registry data from WA Cancer Registry (2013-2017)<br><br>122,402 cases, including malignant and selected benign/uncertain tumours	Rare cancers make up 21.5% of all cancer diagnose in WA, with a significantly poorer five year survival of 58.2% compared to patients diagnosed with a common cancer whose five year survival was 87.8%. Survival for less common cancers was significantly poorer than both rare and common cancers, at 48.1%. Together, rare and less common cancers represent 48.4% of all cancer diagnoses in WA.<br>The results suggest that Aboriginal people with a cancer diagnosis are also more likely to be diagnosed with a rare cancer than non-Aboriginal people.	Improved health service design and delivery for cancer patients in WA, particularly for those with rare and less common cancers.<br>Further research is needed to understand disparities in cancer rates between Aboriginal and non-Aboriginal populations.
Calver et al. (2005) WA [47]	Examine the impact of a multi-component health assessment on mortality and morbidity in Kimberley Aboriginal residents during a 13-year follow up.	Education, communication, and support<br><br>Randomised controlled trial<br><br>620 (intervention), 6,736 (control subjects)	A 13-year follow-up of a health assessment found that while it led to increased initial contact with health services among Kimberley Aboriginal residents, it did not result in significant long-term health improvements.<br>The intervention group experienced higher rates of first-time hospitalizations, including injury-related episodes, and a higher incidence of alcohol-related cancers compared to the control group. No overall mortality benefit was observed, indicating that while health assessments may prompt increased health service use, they do not inherently improve longevity or reduce mortality.	While multi-component health assessments can increase initial health service engagement, they are unlikely to yield long-term health improvements for Indigenous populations.<br>Investments should focus on continuous, comprehensive programs that address broader social determinants of health and integrate community development strategies.

First Author (Year) Location	Study aim	Study Focus, Design and Population	Findings	Recommendations
Carter et al. (2021) WA, Queensland [26]	Investigate the cost-effectiveness of risk-stratified screening for hepatocellular carcinoma (HCC) in the Australian healthcare system, using a serum biomarker test to target resources to high-risk patients.	Screening and prevention<br><br>A decision-analytic modelling study using a Markov cohort model to compare 3 different screening strategies for patients with compensated cirrhosis: risk-stratified screening, all-inclusive screening, and no formal screening.<br><br>Not applicable	A biomarker-based risk- stratified screening approach to detect HCC is likely to be cost effective in the Australian healthcare system relative to the current practice of no formal screening, but further evidence is required to increase the certainty of these findings.<br>When comparing a risk stratified screening approach with an all inclusive approach, the all inclusive approach is more likely to be cost-effective.<br>Both risk stratified and all inclusive screening programs were found to be more favourable for Aboriginal and Torres Strait Islanders.	Cirrhosis population-wide screening for HCC is likely to be cost-effective in Australia.<br>Risk-stratified screening using a serum biomarker test may be cost-effective at lower WTP thresholds.<br>Successful implementation will require a coordinated approach by the Liver Foundation, Cancer Council, community agencies, and state and federal governments and provide public education to vulnerable patients and other geographically isolated populations.
Christou & Thompson (2012) WA [48]	Examine awareness, knowledge, attitudes and behavioural attention regarding bowel cancer screening	Screening and prevention<br><br>Cross-sectional study<br><br>93 Indigenous Australians	Awareness and knowledge of colorectal cancer (CRC) screening were low, although both were significantly associated with exposure to media advertising.<br>Nearly two-thirds (63%) of respondents intended to participate in screening, however 84% said they would participate on a doctor’s recommendation.<br>Predictors of screening intention included: perceived self-efficacy in completing the FOBT test, participating in any cancer screening in the past two years and greater knowledge about bowel cancer.	Ensure that national screening programs account for the social and cultural diversity of the population, including the needs of Indigenous and minority groups.<br>Address the barriers created by the current design of the NBCSP, such as the postal distribution of the FOBT and requirement for self-screening, which disadvantages low-literacy populations and reduces self-efficacy.<br>Develop health promotion and education strategies that focus on improving knowledge, awareness, and self-efficacy around CRC screening among Indigenous Australians
Condon et al. (2014) Australia [33]	Validate analysis methods and report cancer survival rates for Indigenous Australians as the basis for regular national reporting.	Incidence and mortality<br><br>National population-based cohort study<br><br>7,019 Indigenous people and 1,228,573 non-Indigenous people with cancer	Significantly lower survival rates for Indigenous Australians compared to non-Indigenous Australians, especially within the first year post-diagnosis.<br>Indigenous Australians were more likely to have late-stage diagnoses, lower rates of accessing cancer treatments, and higher levels of comorbidities, particularly in rural and remote areas.<br>Survival was lower for those living in remote regions, where Indigenous people experienced a 65% higher mortality rate than those in urban areas.<br>Over time improvements in cancer survival were seen in non-Indigenous populations but only minimally for Indigenous groups, and only in remote areas.	More accurate recording of Indigenous status by cancer registers is needed.<br>Cancer care for Indigenous Australians needs significant improvement.<br>Cancer diagnosis, treatment, and support services should be redesigned to be accessible and acceptable to Indigenous people.<br>Investigate the reasons for the poor survival of cancer cases from rural and remote areas, and how to overcome them.

First Author (Year) Location	Study aim	Study Focus, Design and Population	Findings	Recommendations
Croager et al. (2010) WA [22]	Develop, deliver and evaluate a cancer education course for Indigenous health professionals, as well as increase cancer knowledge and awareness of resources and services for Aboriginal people with Cancer and their families.	Education, communication, and support in cancer care<br><br>A four-day workshop with pre and post evaluation<br><br>35 Aboriginal Health Professionals	A culturally tailored cancer education course significantly increased Aboriginal Health Professionals' knowledge and confidence in cancer prevention, diagnosis, and management immediately after training.<br>At follow-up, only certain confidence aspects, such as understanding cancer basics and identifying common types, were maintained over time, with most knowledge gains diminishing.<br>A short, culturally relevant training course increases cancer knowledge and confidence, however, ongoing education is needed to maintain this.	Provide ongoing education and support for the Aboriginal Health Professionals, such as regular contact, newsletters, facilitator visits, and refresher programs, to maintain their cancer knowledge and confidence.<br>Establish partnership or mentoring opportunities where the Aboriginal Health Professionals can work with local cancer experts to deliver community education on cancer-related issues.
Darcey et al. (2019) WA [27]	Determine if mammographic density is associated with an increased risk of breast cancer in Aboriginal women in WA, similar to findings in other populations.	Screening and prevention<br><br>Retrospective “double” case–control study<br><br>103 Aboriginal women with breast cancer (327 controls) and 341 non-Aboriginal women with breast cancer (333 controls)	Mammographic density is a strong predictor of breast cancer risk in Aboriginal women in WA, similar to its role in non-Aboriginal women.<br>Despite having generally lower mammographic density than non-Aboriginal women, Aboriginal women with higher density were found to have a greater risk of breast cancer compared to their peers with lower density.<br>For each increase in dense breast area or percentage dense area, the odds of developing breast cancer rose significantly in Aboriginal women.	Breast cancer risk prediction models and efforts to standardize risk-associated mammographic density measures should take Aboriginal status into account.<br>Aboriginal women with dense breasts could potentially benefit from supplemental screening using a modality other than mammography.
Diaz et al. (2019) WA, NSW, NT, Queensland, SA [53]	Identify service-level factors associated with variation in cervical screening coverage between Indigenous primary health care centres, and understand the service-level factors underlying this variation in order to identify improvements to increase screening participation.	Screening and prevention<br><br>Cross-sectional clinical audit<br><br>3,801 women (3,378 Indigenous women) attending 135 Indigenous primary health care centres	Substantial variation in cervical screening rates among 135 Indigenous Primary Health Care (PHC) centres.<br>Service-level factors, including longer participation in continuous quality improvement (CQI) programs and centres located in very remote areas, were linked to higher screening coverage.<br>Client-level factors, such as age and recent engagement with the PHC, contributed minimally to the overall differences.<br>Centres with sustained involvement in CQI initiatives had improved delivery of cervical screening services.	Strategies for improving the delivery of cervical screening by Indigenous PHC centres should be devised by, and for, Aboriginal and Torres Strait Islander women.<br>To maximize the benefit of CQI programs, it is necessary to understand how organizational, environmental and system-level factors moderate the relationship between CQI participation and service delivery.

First Author (Year) Location	Study aim	Study Focus, Design and Population	Findings	Recommendations
Doss et al. (2023) NT [54]	Assess the significance and applicability of current guidelines regarding the threshold for abnormal mediastinal lymph node enlargement (MLE) among adult Indigenous Australians.	Incidence and mortality<br><br>Retrospective cohort study<br><br>49 Indigenous adult patients with MLE and with at least two CT scans available	MLE was common among adult Indigenous Australians undergoing chest CT, with most cases presenting nodes larger than 10 mm.<br>The majority of enlarged lymph nodes were stable or benign in nature, with only a small proportion progressing to lung malignancy.<br>Over a median follow-up of nearly two years, 65% of patients had stable MLE, while 35% experienced size changes, either increasing or decreasing. Only three patients were diagnosed with lung cancer.<br>Current guidelines for MLE size thresholds may not fully apply to Indigenous Australians, as the majority of enlarged lymph nodes in this group did not indicate malignancy.	Consider increasing the normal mediastinal lymph node size threshold from 10 mm to 15 mm for Indigenous Australian patients, in the absence of other suspicious findings.<br>Favor clinical and periodic CT surveillance over invasive procedures like EBUS, especially in resource-poor settings where access to EBUS is limited.<br>Conduct long-term prospective studies to better characterize normal lymph node size and changes in Indigenous Australians with neoplastic vs non-neoplastic lung disease.
Franklin et al. (2016) WA [42]	Investigate the incidence of malignant mesothelioma (MM) among Aboriginal people in WA and to identify the primary pathways through which asbestos exposure occurred within this population.	Incidence and mortality<br><br>Descriptive epidemiological study<br><br>39 Aboriginal people in WA with MM	MM incidence among Aboriginal people in WA is significantly associated with asbestos exposure from the Wittenoom mining operations.<br>39 cases of MM were identified among Aboriginal people, with 67% of these cases directly linked to the crocidolite (blue asbestos) mining activities and environmental contamination surrounding Wittenoom. Aboriginal people demonstrated consistently higher 10-year incidence rates compared to non-Aboriginal people, with MM cases in this group more commonly resulting from non-occupational exposure than in non-Aboriginal cases.<br>Aboriginal cases of MM were diagnosed at a younger age, reflecting both earlier exposure and shorter life expectancy within this population.	None
Haggar et al. (2013) WA [56]	Evaluate survival and excess mortality among adolescents and young adults (AYAs) with cancer in WA from 1982 to 2004, using population-based data from the Western Australian Data Linkage System.	Incidence and mortality<br><br>Population based retrospective observational study<br><br>10,266 AYAs (821 Indigenous) with malignant neoplasms	Survival rates for AYAs diagnosed with cancer in WA improved significantly over the study period from 1982 to 2004.<br>Aboriginal AYAs faced higher excess mortality compared to non-Aboriginal counterparts, especially for germ cell tumours.<br>Those residing in rural or remote areas and those from lower socioeconomic backgrounds had increased mortality risk.<br>Cancer type also played a critical role in survival, with melanoma and germ cell tumours showing the most favourable outcomes, whereas central nervous system	Further research into the causes of survival disparities, including differences in tumour biology and healthcare access.<br>Promote equity in service access and consider novel therapeutic strategies for certain cancers.

First Author (Year) Location	Study aim	Study Focus, Design and Population	Findings	Recommendations
			malignancies and leukemias were associated with poorer survival rates.	
Hall et al. (2004) WA [59]	Examine possible disparities in the uptake of cancer surgery for breast, prostate, and lung cancer between the Indigenous and non-Indigenous populations in WA	Barriers and disparities<br><br>Epidemiological survey<br><br>13,377 lung cancer patients (274 Indigenous)<br>14,187 prostate cancer patients (64 Indigenous)<br>11,206 women who had breast cancer surgery (151 Indigenous)	Results indicate a different pattern of surgical care for Indigenous patients in relation to lung and prostate cancer, but not breast cancer.<br>Indigenous patients with lung or prostate cancer were less likely to receive surgical treatment compared to non-Indigenous patients.<br>Indigenous women with breast cancer had similar rates of breast-conserving surgery compared to non-Indigenous women.<br>Lower cancer incidence rates in the Indigenous population may be partly due to underreporting.<br>Indigenous cancer patients may have higher case fatality rates, potentially due to higher incidence of cancers with high fatality, later diagnosis, or suboptimal treatment patterns.	Investigate reasons for disparities in treatment choice and barriers to care.<br>Focus on disparities in surgical care for Indigenous patients compared to non-Indigenous patients.
Hall et al. (2004) WA [58]	Examine patterns of in-hospital surgical care for lung cancer in WA, focusing on how demographic, locational, and socio-economic factors, as well as private health insurance status, influenced the likelihood of patients receiving surgery.	Barriers and disparities<br><br>Retrospective cohort study<br><br>12,708 lung cancer patients (269 Indigenous)	Only 16% of lung cancer patients in WA received surgical treatment, with significant disparities based on demographic and socio-economic factors.<br>Younger, female, and non-Indigenous patients, as well as those with less comorbidity, were more likely to undergo surgery.<br>Surgical intervention was notably lower among patients from socio-economically disadvantaged backgrounds, particularly those who had their first hospital admission for lung cancer in rural hospitals, where the odds of surgery were significantly diminished.<br>Patients with private health insurance had a higher likelihood of receiving surgery compared to those treated as public patients.	Policies aimed at earlier prevention, diagnosis, and referral for lung cancer.<br>Education of rural doctors to address "nihilistic attitudes" towards lung cancer.<br>Fast-tracking of patients from rural and indigenous backgrounds to multidisciplinary teams in metropolitan centres.<br>Improving cultural awareness and removing barriers to treatment access for indigenous patients.<br>Prioritizing earlier diagnosis, especially in disadvantaged groups.<br>Encouraging people to see their GP earlier in response to symptoms, rather than screening.<br>Evaluating new screening methods like CT scans for Indigenous smokers.<br>Continue efforts by health promoters and health care workers to reduce the prevalence of smoking in the community.
Jessop et al. (2021) WA, NT, SA [31]	Compare the presentation patterns, follow-up, and clinical	Barriers and disparities<br><br>Retrospective cohort	Significant disparities were observed in treatment and outcomes for Indigenous children with acute leukaemia compared to non-Indigenous children.	Effective strategies are required to ensure children from regional/rural locations and Indigenous children with leukaemia receive appropriate

First Author (Year) Location	Study aim	Study Focus, Design and Population	Findings	Recommendations
	outcomes of Indigenous and non-Indigenous children with acute leukemia in Australia, and to assess the impact of remoteness and area-based socioeconomic disadvantage on outcomes.	study<br><br>455 children treated for acute leukaemia (29 Indigenous)	Indigenous children had lower rates of enrolment in clinical trials (4.2% vs. 53.1% for non-Indigenous) and a higher rate of loss to follow-up (26.1% vs. 9.2%).<br>Geographic remoteness was associated with inferior overall survival for all children with leukaemia.	service delivery and resource allocation to improve their survival and follow-up.
Luke et al. (2022) WA, NT, Queensland [36]	Investigate access to clinical genetic health services among Aboriginal and Torres Strait Islander people by examining appointment scheduling and attendance rates compared to non-Indigenous populations.	Barriers and disparities<br><br>Cross-sectional observational study<br><br>14,870 people (654 Indigenous) with an appointment scheduled at one of three clinical genetic health services between 2014-2018	Significant disparities were observed in access to clinical genetic health services for Aboriginal and Torres Strait Islander people compared to non-Indigenous populations.<br>Aboriginal and Torres Strait Islander individuals were scheduled for fewer appointments and attended at lower rates.<br>Disparities were most pronounced among adults, females, residents in remote areas, and those referred for cancer or prenatal reasons.	Targeted interventions to enhance access to clinical genetic health services for Aboriginal and Torres Strait Islander populations.<br>Improve referral pathways and increase awareness of genetic services among primary care providers to ensure equitable access, especially for adults and those in remote areas.<br>Develop culturally safe and responsive care models that incorporate the input of Aboriginal and Torres Strait Islander communities to address specific needs and preferences.<br>Enhance telehealth services to reach underserved populations.<br>Improve data collection practices to ensure accurate representation of Aboriginal and Torres Strait Islander status in health databases.
McLean et al. (2019) WA [63]	Compare the distributions and determinants of mammographic density (MD) between Aboriginal and non-Aboriginal women in WA.	Screening and prevention<br><br>Cross-sectional study<br><br>628 Aboriginal women and 624 non-Aboriginal women	Aboriginal women exhibited lower absolute dense area (DA) and percent dense area (PDA) compared to non-Aboriginal women, indicating that they have, on average, less mammographic density.<br>Age and several socio-economic indices were significantly associated with mammographic density measures for both populations, suggesting that as age increases, mammographic density decreases.<br>Remoteness of residence was linked to mammographic measures, particularly among non-Aboriginal women.<br>The lower mammographic density in Aboriginal women may improve the sensitivity of mammographic screening, which is particularly crucial given the higher breast cancer mortality rates in this population.	Culturally sensitive promotion of the benefits of mammographic screening be implemented to improve participation rates among Aboriginal women, who tend to have lower mammographic density.<br>Further research be conducted to explore the underlying reasons for the lower mammographic density in Aboriginal women and to investigate the relationship between mammographic density measures and breast cancer risk in this group.<br>Increased community awareness and access to screening services, along with targeted educational initiatives, could help address the current barriers to participation in mammographic screening within Aboriginal communities.

First Author (Year) Location	Study aim	Study Focus, Design and Population	Findings	Recommendations
Ngweso et al. (2023) WA [64]	Review the mortality of patients diagnosed with penile cancer in WA between 1992 and 2017 and determine if Aboriginal people and patients in rural and remote regions experience discrepancies in survival outcomes.	Incidence and mortality<br><br>Retrospective cohort study<br><br>186 patients with penile cancer (9 Aboriginal)	Aboriginal people with penile cancer and men from regional or remote areas of WA experience significantly worse penile cancer-specific survival outcomes compared to their non-Aboriginal counterparts and those living in urban settings.<br>Aboriginal patients had a hazard ratio of 6.512, indicating they were at a much higher risk of penile cancer-specific mortality.<br>55% of the patients who died from penile cancer were from outer regional or remote areas, highlighting the substantial disparities in survival rates associated with geographic and demographic factors.	Enhance public health measures, educational resources, and stakeholder engagement to address health discrepancies and improve outcomes.<br>A focus on improving data accuracy and healthcare accessibility is crucial.<br>Increase awareness about penile cancer among the general population and clinicians, potentially through improved evidence-based internet resources.<br>Investigate barriers to care and quality of life impacts, especially in Aboriginal and rural/remote populations.<br>Further research should explore the clinical and pathological characteristics of the disease, treatment disparities, and their implications on survival.
Raymond et al. (2023) WA [65]	Explore the risk of cancer and 5-year mortality in patients with systemic lupus erythematosus (SLE) in WA.	Incidence and mortality<br><br>Population level, data linkage study<br><br>2,111 patients with SLE (165 Aboriginal) and 21,110 comparators (1,275 Aboriginal)	Hospitalized patients with SLE had a similar risk of cancer development compared to the general population.<br>Aboriginal Australians had lower age- and sex- adjusted risk of incident overall cancer development; due to lower risk of colorectal, breast (female only), and skin cancer.<br>Aboriginal Australians had higher risk of incident cancer development of the hepatobiliary, respiratory (lung), musculoskeletal, and female reproductive systems.<br>A SLE- related hospitalization conferred no additional risk of cancer development of Aboriginal Australians.	Healthcare providers implement enhanced cancer prevention and surveillance strategies specifically tailored for patients with SLE. This includes regular screening for high-risk cancers, particularly oropharyngeal, vulvo-vaginal, and skin cancers, given the increased risk observed in younger SLE patients.<br>Education on the importance of sun protection and lifestyle modifications, such as smoking cessation, should be emphasized to mitigate further risks.<br>Healthcare systems should consider integrating multidisciplinary care approaches that involve rheumatologists, oncologists, and primary care providers to ensure comprehensive management of SLE patients, particularly those with comorbidities that may exacerbate cancer risk.

First Author (Year) Location	Study aim	Study Focus, Design and Population	Findings	Recommendations
Rosenwax & McNamara (2006) WA [66]	Describe the characteristics of people in WA who received, and did not receive, specialist palliative care (SPC) during their last 12 months of life between 2000 and 2002.	Barriers and disparities<br><br>Retrospective cohort study<br><br>26,882 deaths, with 7,399 deaths from cancer (98 Aboriginal), 608 deaths from both cancer and non-cancer conditions, and 6,712 deaths from selected non-cancer conditions. (182 Aboriginal)	Significant disparities in access to specialist palliative care (SPC) among individuals who died in WA between 2000 and 2002.<br>Two-thirds (68%) of people who died of cancer received SPC, only 8% of those who died from selected non-cancer conditions accessed these services.<br>Individuals who were single or widowed, aged 85 years or older, or living outside major urban areas were less likely to receive SPC.<br>Indigenous people who died of cancer were more likely to have used SPC.<br>Indigenous people who died from selected non-cancer conditions were less likely to have accessed SPC, although this finding was not statistically significant.	Extend SPC services to people with non-cancer conditions, as they are currently underserved compared to cancer patients.<br>Improve access to SPC for cancer patients as well, as a significant proportion still do not receive it.<br>Provide more culturally appropriate, community-based SPC services for Indigenous patients, rather than relying on hospital-based care.
Spilsbury et al. (2006) WA [70]	Investigate incidence trends and identify the demographic, social, and health factors associated with the rate of hysterectomy and morbidity outcomes in WA.	Treatment and outcomes<br><br>Retrospective cohort study<br><br>83,068 hysterectomy procedures performed on women aged 20 years and older in WA from 1981 to 2003.	Age-standardized rate of hysterectomy in WA decreased by 23% from 1981 to 2003, although it remained one of the highest in the world.<br>The lifetime risk of undergoing a hysterectomy was estimated at 35%.<br>Women from rural and remote areas, as well as those with lower socioeconomic status and public health insurance, were more likely to undergo hysterectomy, particularly for menstrual disorders.<br>Indigenous women had lower overall hysterectomy rates compared to non-Indigenous women but had higher rates of hysterectomy for gynaecological cancers, particularly in rural areas.	None
Subramaniam et al. (2005) WA [71]	Assess the incidence, age, sex, and Indigenous distribution of hospital separations for oral malignancies in WA over a four-year period from 1999/2000 to 2002/2003.	Incidence and mortality<br><br>Retrospective cohort study<br><br>3,747 patients hospitalized for oral malignancies (80 Aboriginal)	An average age of 58 years at the time of hospitalization.<br>Males were hospitalized at nearly twice the rate of females.<br>Indigenous Australians had a 1.3 times higher rate of hospitalization for oral malignancies compared to non-Indigenous individuals.<br>Indigenous patients experienced a 3.5 times higher rate of palate malignancies and a 1.4 times higher rate of tonsil malignancies.<br>Indigenous patients spent nearly twice as long in hospital compared to non-Indigenous patients.	Focus on prevention by limiting exposure to known risk factors for oral malignancies.<br>Address the increasing impact of oral malignancies on the aging population, as they will impose a significant demand on the health resources.<br>Promote the importance of prevention and early detection of oral malignancies to both the community and healthcare professionals.<br>Conduct further research on the impact of early screening and prevention programs, especially for certain types of oral cancers, to reduce the burden on the healthcare system.

First Author (Year) Location	Study aim	Study Focus, Design and Population	Findings	Recommendations
Taylor et al. (2018) Australia [72]	Examine where Indigenous Australians undergo cancer treatment and learn about specific service initiatives that have been implemented to meet their needs.	Treatment and outcomes<br><br>Descriptive cross-sectional survey<br><br>58 public cancer treatment centres	Indigenous cancer patients represented a small proportion of the overall patient load in most participating centres, with less than 5% of total patients being Indigenous in the majority of cases.<br>About 47% of the centres reported seeing more than 10 Indigenous patients annually.<br>The most common initiatives reported were having links with Indigenous health organisations (74%), making a dedicated effort to address the needs of Indigenous patients (69%), and creating partnerships with Indigenous communities (69%).<br>Fewer respondents (58%) indicated that they had established specific programs or services for Indigenous patients and their families, and only 55% had policies guiding interactions with Indigenous patients.	None
Valery et al. (2013) Australia [39]	Assess the variation in childhood cancer survival rates between Indigenous and non-Indigenous Australian children. .	Incidence and mortality<br><br>Population-based cohort study<br><br>6,572 children (196 Indigenous children)	The five-year survival rate for Indigenous children diagnosed with cancer was 75.0%, lower than the 82.3% for non-Indigenous children.<br>Indigenous children had 1.36 times the risk of dying within five years of diagnosis after adjusting for factors such as geographic remoteness, socio-economic disadvantage, cancer type, and year of diagnosis.<br>The survival gap was most pronounced for "other tumours" (e.g., lymphomas and neuroblastomas), where Indigenous children were 1.83 times more likely to die compared to non-Indigenous children.<br>Indigenous children in remote areas and those from socio-economically disadvantaged regions faced even greater survival challenges.	Investigation of cancer treatment and compliance are needed as they could also potentially be contributing factors to the disparity in cancer survival among children with certain cancers.<br>Better understanding of the patterns of care will help to identify factors where modification may improve outcomes.
White et al. (2011) WA [3]	To compare the unmet supportive-care needs of rural/remote and metropolitan cancer patients in Western Australia	Education, communication, and support<br><br>Cross-sectional study<br><br>786 cancer patients (7 Aboriginal)	The unmet needs of cancer patients did not differ significantly between metropolitan, rural, and remote areas of Western Australia, contrary to expectations.<br>Informal support structures in rural communities may help to reduce the unmet needs of rural cancer patients.	Use the study's findings to guide the development of interventions to address the unmet needs of cancer patients, particularly those in remote and rural areas.<br>Conduct a survey of the unmet needs of rural and remote carers of cancer patients to ensure that cancer services are meeting the needs of all individuals involved in a patient's cancer journey.
