# Supplementary material for: A Review of Western Australian Researchers’ Contributions to Understanding Cancer Prevention and Outcomes in Aboriginal People
Source: Int J Environ Res Public Health. 2026 Jun 10;23(6):777. doi: 10.3390/ijerph23060777 (PMC13300084; doi:10.3390/ijerph23060777)
Supplement: Supplementary file 1 [file ijerph-23-00777-s001.zip › Supplementary File S4(ReviewArticles).pdf]

First Author (Year)	Study aim	Study Focus and Design	Findings	Recommendations
Christou et al. (2010) [48]	Analyse Australia's National Bowel Cancer Screening Program (NBCSP) to identify barriers to participation by Indigenous Australians and ethnic minorities.	Screening and prevention<br><br>Systematic review	The NBCSP is not reaching many Indigenous Australians in the target group. Factors contributing to lower participation include how participants are selected, the way the screening kit is distributed, the nature of the test and comprehensiveness of its contents, cultural perceptions of cancer and prevailing low levels of knowledge and awareness of bowel cancer and the importance of screening.	Modify the NBCSP to be more accessible and tailored to the needs of Indigenous Australians and other minority groups, through a more community-based and primary care-integrated approach.<br>Provide alternative mechanisms for FOBT test kit delivery and return.<br>Ensure dedicated health workers to provide personalized support and assistance with completing the test.<br>Integrate screening into primary care and chronic disease management.<br>Improve data collection on Indigenous participation and follow-up.<br>Increase availability of culturally appropriate education materials and promotion of the program.<br>Further research to understand the needs and social and cultural sensitivities of Indigenous and other minority populations around cancer screening and to inform alternative approaches to bowel cancer screening.<br>Ensure Indigenous Australians have access to FOBT kits from the age of 40-45 years.
Clark et al. (2024) [32]	Advance precision cancer medicine specifically for Indigenous Australians.<br>Develop requirements to inform and guide HPs, researchers and funders on precision cancer medicine as it applies to Indigenous Australians.	Treatment and outcomes<br><br>Narrative review	Indigenous Australians are under-represented in genomics and cancer studies; significant genomic variation exists.<br>Need for culturally appropriate genome reference resources; need for Indigenous-led research and governance; importance of community engagement and ethical guidelines	Equity in access to care and clinical trials<br>Indigenous-specific genome reference resources, as the unique genomic variations in these populations affect diagnosis and treatment options.<br>An Indigenous cancer workforce<br>Indigenous data sovereignty and Indigenous governance in cancer research.<br>Community-driven research and policies to ensure culturally relevant and impactful cancer care solutions.<br>Local and national genomic health research guidelines are needed to define best practice in genomics research with Indigenous Australians.

First Author (Year)	Study aim	Study Focus and Design	Findings	Recommendations
Davidson et al. (2012) [51]	Examine the experience of lung cancer among Indigenous Australians, focusing on identifying reasons for poorer outcomes and lower levels of treatment compared to non-Indigenous Australians.<br>Explore opportunities for early intervention and improvement in policy, service planning, and healthcare delivery to address these disparities effectively.	Barriers and disparities<br><br>Integrative review	High smoking rates, cultural beliefs, geographic remoteness, socioeconomic disadvantages, and systemic racism create substantial barriers to accessing timely and effective lung cancer care.<br>Many Aboriginal Australians experience late-stage diagnoses and receive less aggressive treatment than non-Indigenous Australians, further impacted by limited specialist services in rural areas, financial burdens, and family obligations that often hinder relocation for treatment.<br>Cultural beliefs, such as perceptions of cancer as a "death sentence" or punishment, alongside mistrust in healthcare institutions due to historical discrimination, also deter early intervention and adherence to treatment.	Improve data collection and identification of Aboriginal populations to better understand the lung cancer burden.<br>Adopt a national policy approach to address lung cancer disparities, with Aboriginal ownership, participation and control.<br>Implement culturally appropriate smoking cessation programs and health literacy strategies.<br>Ensure mainstream healthcare reflects the impact of colonization and considers alternative beliefs/healing methods, and develops tailored lung cancer prevention and care strategies.<br>Address health disparities through coordinated policy initiatives across health and social policy, with effective engagement and partnership with Aboriginal communities
Erber et al. (2004) [55]	Investigate the unique haematological characteristics of Indigenous Australians, including inherited and acquired factors influencing the blood profiles, blood group distributions, and the incidence of various haematological conditions within this population.	Incidence and mortality<br><br>Systematic review	Unique haematological characteristics exist among Indigenous Australians including: variation in blood group frequencies compared to non-Indigenous populations; an absence of factor V Leiden and prothrombin gene mutations in Indigenous populations, which are typically associated with thrombotic risk in other groups; and high prevalence of alpha-thalassaemia, which impacts red blood cell structure.<br>Lifestyle and environmental factors, such as dietary changes, parasitic infections, and substance abuse, also play a role in acquired haematological variations, including eosinophilia and macrocytic anaemia.	Further research to confirm the reported low incidence of haematological malignancies compared with non-Indigenous Australians.
Haigh et al. (2018) [57]	Provide an overview of key information on cancer among Indigenous Australians, including incidence, prevalence and survival data; mortality and burden of disease; health service utilisation; cancer prevention; and programs, services and polices to address cancer among Indigenous Australians.	Treatment and outcomes<br><br>Narrative review	Indigenous Australians face significant disparities in cancer incidence, diagnosis, and outcomes compared to non-Indigenous populations.<br>Indigenous people are often diagnosed at more advanced stages of cancer, leading to poorer survival rates and higher mortality.<br>While some types of cancer have lower incidence rates among Indigenous Australians, those with higher risks, such as lung, liver, and cervical cancers, are often associated with lifestyle factors like smoking and chronic infections.	Improved access to culturally appropriate healthcare services that align with the unique needs of Indigenous communities.<br>Enhanced education and health promotion efforts to increase awareness about cancer prevention and early detection.<br>Develop targeted anti-smoking and alcohol-reduction programs, given their strong link to cancers prevalent among Indigenous people, such as lung and liver cancer.<br>Comprehensive data collection and improved reporting on Indigenous status in health registries.<br>Community participation in healthcare delivery, ensuring services are culturally sensitive and trust-building.

First Author (Year)	Study aim	Study Focus and Design	Findings	Recommendations
Ireland et al. (2023) [60]	Evaluate interventions aimed at increasing bowel cancer screening uptake among Indigenous populations, particularly in rural and remote communities.	Screening and prevention<br><br>Scoping review	Nineteen strategies to increase bowel cancer screening participation were identified including recommendation from a GP, culturally appropriate education resources and following up of non-responders. Strategies specific to rural and remote communities included alternative distribution of kits and mobile screening.	Focus on the most effective strategies identified, including GP endorsement, culturally appropriate education, and follow-up with non-responders.<br>Prioritize alternative distribution of screening kits and mobile screening services to increase Indigenous population screening in rural and remote communities.<br>Tailor the strategies to the specific needs and barriers within each region or community.
Koefler (2012) [61]	Investigate the reasons why Indigenous Australians experience significantly higher cancer mortality rates compared to non-Indigenous Australians.	Barriers and disparities<br><br>Literature review	Indigenous Australians have significantly poorer cancer outcomes compared to non-Indigenous Australians, with a higher mortality rate after adjusting for age, cancer type, and stage at diagnosis.<br>Indigenous Australians face a higher incidence of smoking-related cancers (such as lung and oropharyngeal cancers) and cancers that are amenable to screening (such as cervical cancer).<br>Contributing factors include late-stage diagnosis, lower rates of curative treatment, and barriers to accessing healthcare services.<br>Barriers to treatment include socioeconomic disparities, geographical isolation, communication difficulties due to language diversity, and non-biomedical cultural beliefs about cancer.	Culturally appropriate healthcare services that are tailored to meet the needs of Indigenous communities.<br>Increased primary health care services.<br>Educational programs aimed at reducing behavioural risk factors such as smoking and improving health literacy, ensuring that these programs are designed with cultural considerations in mind.<br>Expand access to screening and preventative services, such as the "Well Women's Screening Program".<br>Greater community engagement by supporting Indigenous cancer survivors as advocates and incorporating their experiences into healthcare planning.
Taylor et al. (2022) [28]	Provide a historical overview of cancer treatment services, screening and end-of-life care in the NT over the past twenty years, with a focus on implications for the NT's Indigenous population.	Treatment and outcomes<br><br>Narrative review	There have been significant improvements in cancer care services in the NT, including the establishment of the Alan Walker Cancer Care Centre (AWCCC) in Darwin, and the arrival a PET Scanner, which have enhanced access to cancer diagnosis and treatment.<br>Telehealth and the establishment of chemotherapy units in remote hospitals have reduced travel requirements for patients within the NT. Obstacles such as cultural disconnects between health services and Indigenous patients, limited accommodation for cancer patients, and issues recruiting and retaining HPs remain.	Improve travel assistance by revising the 200 km eligibility limit for the Patient Assistance Travel Scheme (PATS) to better accommodate rural patients.<br>Increase the availability of suitable accommodations for patients traveling for treatment.<br>Develop additional strategies to recruit and retain the cancer workforce, particularly non-Indigenous and Indigenous health professionals working in remote settings.<br>Develop localized strategies to increase participation in cancer screening programs in collaboration with the local Indigenous communities.<br>Develop increased cancer education for remote health professionals.

First Author (Year)	Study aim	Study Focus and Design	Findings	Recommendations
Tranberg et al. (2016) [30]	Identify the factors influencing cancer treatment decision-making among Indigenous peoples.	Treatment and outcomes<br><br>Systematic review	Socio-economic and cultural factors that had the potential to influence cancer treatment decision-making among Indigenous people were grouped into four themes: Spiritual beliefs, encompassing fatalistic views about cancer as a death sentence; Cultural influences, emphasizing the importance of family and community in decision-making and the shame associated with cancer diagnoses; Communication, highlighting the barriers Indigenous individuals face in discussing cancer with healthcare professionals and family members; and Existing healthcare systems and structures, which reflect the challenges of accessing appropriate care, particularly for those in rural or remote areas.	HPs should acknowledge and respect the spiritual and cultural beliefs of Indigenous peoples regarding cancer.<br>Train HPs in interpersonal skills to foster therapeutic relationships that engage and empower Indigenous patients throughout their treatment journeys.<br>Incorporate Indigenous perspectives into health education programs to address misconceptions about cancer and its treatment.<br>Cancer treatment plans for Indigenous patients should recognize the central role of connection to family, community, and land.<br>Enhance access to culturally relevant support services and utilizing community-based health positions to facilitate better communication and linkages between patients and health services.<br>Further research is needed on: investigating cancer treatment decision-making among Indigenous peoples, health professionals' knowledge of and attitudes towards Indigenous people's spirituality and 'best practice' in HP communication with Indigenous people.
Van Schaik & Thompson (2012) [19]	Explore Indigenous beliefs about cancer treatment and treatment efficacy, including 'bush medicine' and biomedical approaches.	Education, communication, and support<br><br>Literature review	Indigenous beliefs about the efficacy of cancer treatments were grouped into five themes: concerns regarding the toxicity and side effects of biomedical treatments; a disconnect between Indigenous patients and HPs often resulting in miscommunication and mistrust; fears related to the need for relocation to urban hospitals for treatment; differing beliefs about disease aetiology; and biomedical treatments failing to address holistic health.	Actively involve Indigenous patients in the design and process of their care, recognizing the importance of cultural sensitivity and the value of Indigenous perspectives.<br>Ensure that patients are truly informed about their treatment options, emphasizing clear communication and understanding to facilitate informed consent.<br>Consider the practical challenges faced by Indigenous patients, such as transportation and separation from family, when developing treatment plans.<br>Understand individual patients' beliefs about cancer and acknowledge holistic views of health, which may include the use of bush medicine.
