# Supplementary material for: A Review of Western Australian Researchers’ Contributions to Understanding Cancer Prevention and Outcomes in Aboriginal People
Source: Int J Environ Res Public Health. 2026 Jun 10;23(6):777. doi: 10.3390/ijerph23060777 (PMC13300084; doi:10.3390/ijerph23060777)
Supplement: Supplementary file 1 [file ijerph-23-00777-s001.zip › Supplementary File S5(MixedMethodsArticles).pdf]

First Author (Year) Location	Study aim	Study Focus, Design and Population	Findings	Recommendations
Christou & Thompson (2013)<br>WA [49]	Evaluate the appropriateness, usefulness, and usage of a culturally relevant educational flipchart for Aboriginal people regarding bowel cancer screening.	Screening and prevention<br><br>Post intervention survey of n=37 flipchart recipients, including n=14 AHPs.	Despite positive feedback on the flipchart's design it was underutilized. Only one-third of respondents had actually used it.<br>Reasons for underutilization included the perception that Aboriginal health education should be handled by AHPs, HPs not prioritising bowel cancer education and lack of opportunity or time.	Greater recognition by all HPs of their potential role in Aboriginal health education.<br>Need for awareness, time, and specific training for effective tool implementation.<br>Training or workshops on health educational resources are crucial for understanding and proper use.<br>Sustained commitment to evidence-based screening programs and inclusion of minority groups are necessary.
Darcey et al. (2019)<br>WA [50]	Assess the feasibility of integrating voluntary height and weight measurements into the routine mammographic screening process within the BreastScreen WA program to support research on the association between BMI and breast cancer risk.	Screening and prevention<br><br>Pilot study.<br>Survey of n=156,072 women, including n=1,833 Aboriginal women.	Incorporating height and weight measurements into routine mammographic screening was feasible, with a 76% participation rate.<br>Participation was lower among Aboriginal women (64%), women with disabilities, and those from non-English-speaking backgrounds, while those from higher socioeconomic backgrounds and less remote areas participated more.<br>Measurements were found to be reliable. Qualitative feedback indicated general support.	BreastScreen WA should adopt voluntary, self-reported height and weight data collection at the time of routine mammographic screening.
Durey et al. (2017)<br>WA [11]	Evaluate whether a workshop on respecting cultural differences improved the confidence of radiation oncology HPs in their knowledge, communication, and ability to offer culturally safe healthcare to Aboriginal cancer patients.	Education, communication, and support<br><br>Pre and post workshop online surveys, with n=39 HP participants.	A single culturally focused workshop significantly enhanced the confidence of radiation oncology health professionals in providing culturally safe care to Aboriginal Australians with cancer.<br>Participants reported increased confidence in engaging respectfully, building trust, understanding the cultural and social contexts of Aboriginal patients, and collaborating effectively with Aboriginal and non-Aboriginal colleagues.<br>Improvements were sustained in most areas even two months after the workshop.<br>Participants displayed a newfound willingness to reflect on their own biases and assumptions, which positively influenced their approach to patient care.	Integrate culturally focused workshops into ongoing professional development for HPs, particularly those working with Aboriginal patients.<br>Establish partnerships between Aboriginal and non-Aboriginal stakeholders and seek feedback from the Aboriginal community on their experiences of care, to improve the care offered to Aboriginal patients with cancer.<br>Expand culturally respectful care training to more healthcare settings and professions.
